# Supplementary material for: Antiviral drug screen identifies DNA-damage response inhibitor as potent blocker of SARS-CoV-2 replication
Source: Cell Rep. 2021 Mar 18;35(1):108940. doi: 10.1016/j.celrep.2021.108940 (PMC7969873; doi:10.1016/j.celrep.2021.108940)
Supplement: Document S1. Figures S1–S5 and Table S2 [file mmc1.pdf]

Cell Reports, Volume *35*

Supplemental information

Antiviral drug screen identifies DNA-damage response inhibitor as potent blocker of SARS-CoV-2 replication

Gustavo Garcia Jr., Arun Sharma, Arunachalam Ramaiah, Chandani Sen, Arunima Purkayastha, Donald B. Kohn, Mark S. Parcells, Sebastian Beck, Heeyoung Kim, Malina A. Bakowski, Melanie G. Kirkpatrick, Laura Riva, Karen C. Wolff, Brandon Han, Constance Yuen, David Ulmert, Prabhat K. Purbey, Phillip Scumpia, Nathan Beutler, Thomas F. Rogers, Arnab K. Chatterjee, Gülsah Gabriel, Ralf Bartenschlager, Brigitte Gomperts, Clive N. Svendsen, Ulrich A.K. Betz, Robert D. Damoiseaux, and Vaithilingaraja Arumugaswami

SUPPLEMENTARY FIGURES

Figure S1

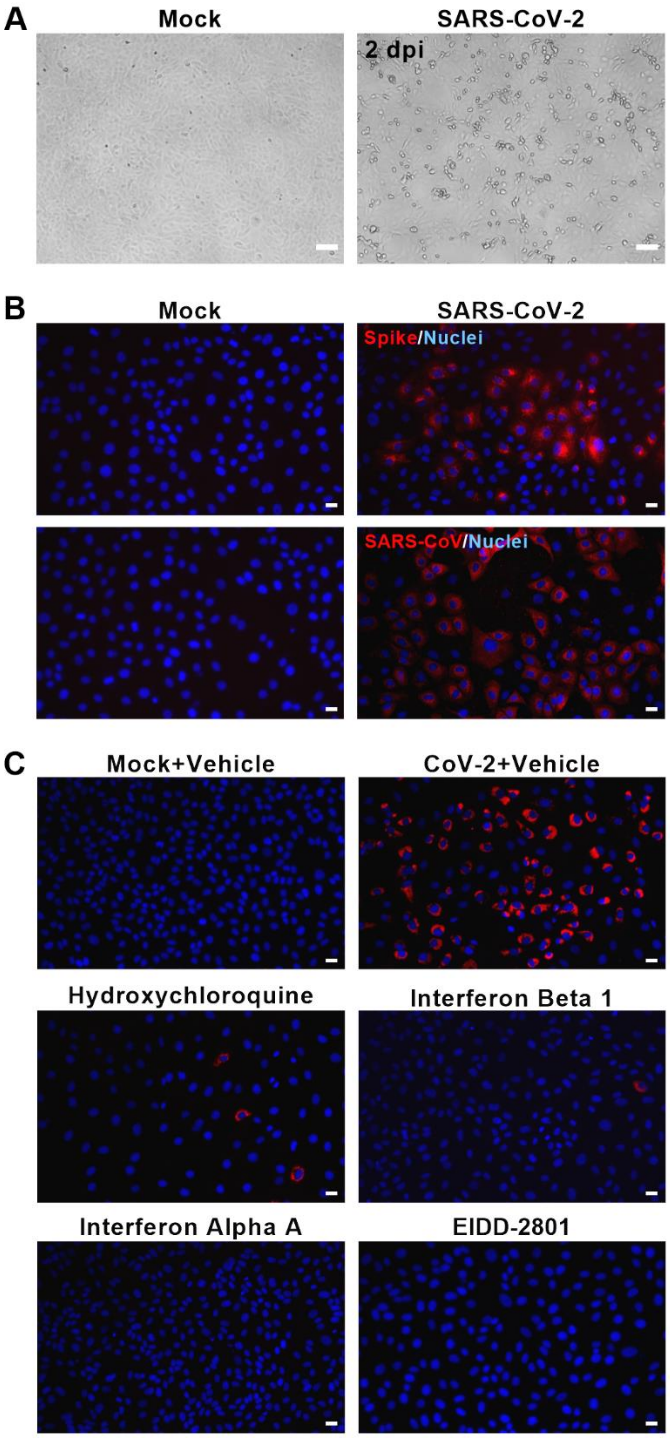

Figure S1. Infectious SARS-CoV-2 cell culture system. (A) Bright field images of Vero E6 cells infected with SARS-CoV-2 virus. Viral cytopathic effects (CPE) are noted in the infected culture. Scale Bar: 50 µm. (B) IFA images of infected cells (48 hpi) stained for SARS-CoV-2. Mouse monoclonal antibody (MS Ab) targeting Spike and a Guinea pig polyclonal SARS-CoV antibody were used. Mock infected cells were included as negative control. Scale Bar: 25 µm. (C) IFA images show compounds with antiviral activity. Images depict that hydroxychloroquine, interferons, and EIDD-2801 (Molnupiravir) (10µM) are effective in blocking SARS-CoV-2 infection. The mock and infected cells were immunostained with dsRNA antibody, which recognizes double stranded genomic RNA generated during viral replication. 20X magnification. Representative data from three or more independent experiments are presented. Related to Figure 1.

Figure S2

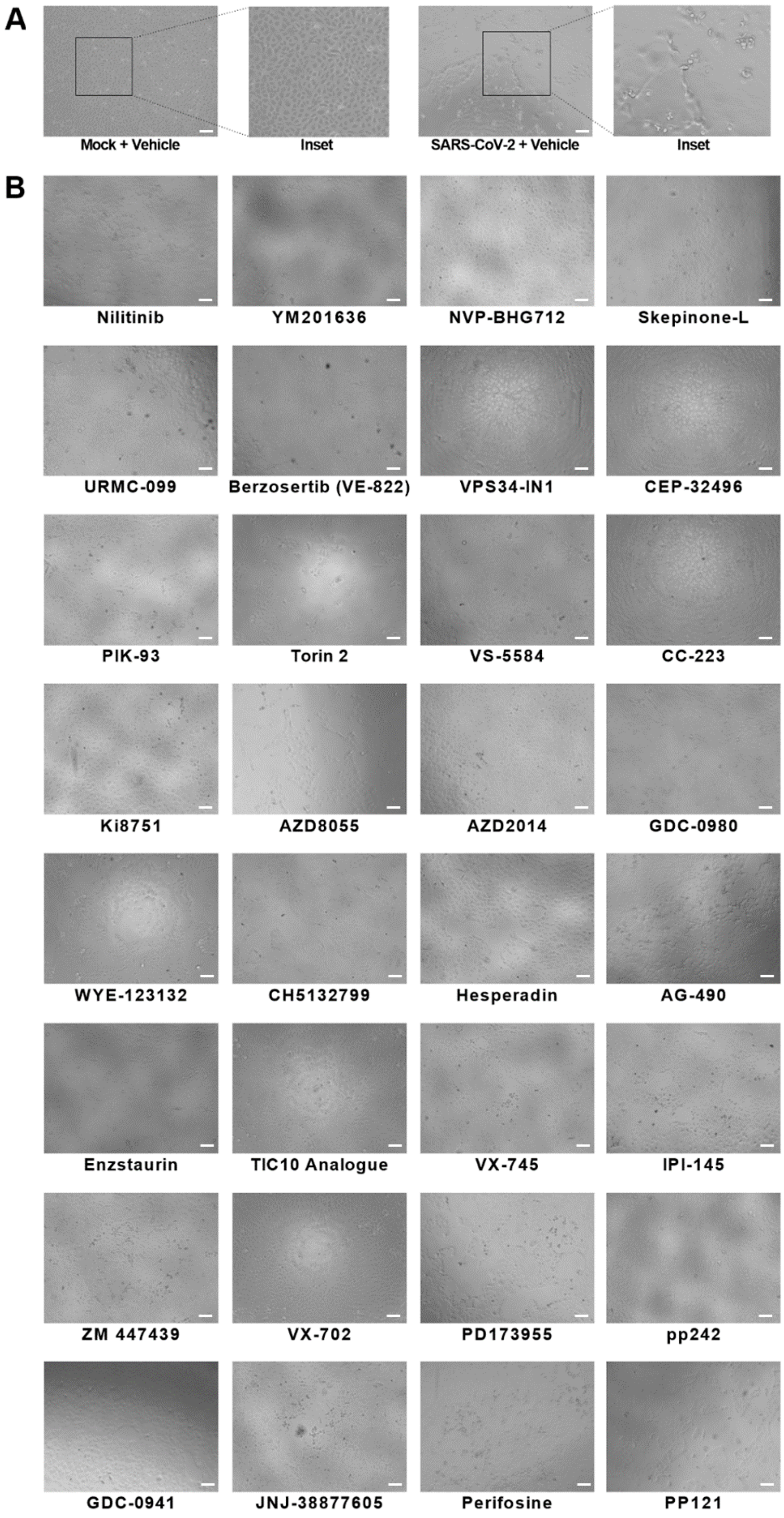
A
Mock + Vehicle
Inset
SARS-CoV-2 + Vehicle
Inset
B
Nilitinib
YM201636
NVP-BHG712
Skepinone-L
URMC-099
Berzosertib (VE-822)
VPS34-IN1
CEP-32496
PIK-93
Torin 2
VS-5584
CC-223
Ki8751
AZD8055
AZD2014
GDC-0980
WYE-123132
CH5132799
Hesperadin
AG-490
Enzstaurin
TIC10 Analogue
VX-745
IPI-145
ZM 447439
VX-702
PD173955
pp242
GDC-0941
JNJ-38877605
Perifosine
PP121

Figure S2. Primary screen of compounds inhibiting SARS-CoV-2 viral cytopathic effect. (A) DMSO Vehicle treated Vero E6 cells with SARS-CoV-2 infection had pronounced viral CPE at 48 hpi. Uninfected cells (Mock+vehicle) are included as negative control. Scale Bar: 50 µm. (B) Bright field microscopic images of drug compounds treated SARS-CoV-2 infected cells showing no or reduced level of viral CPE. Scale Bar: 50 µm. Related to Figure 1.

Figure S3

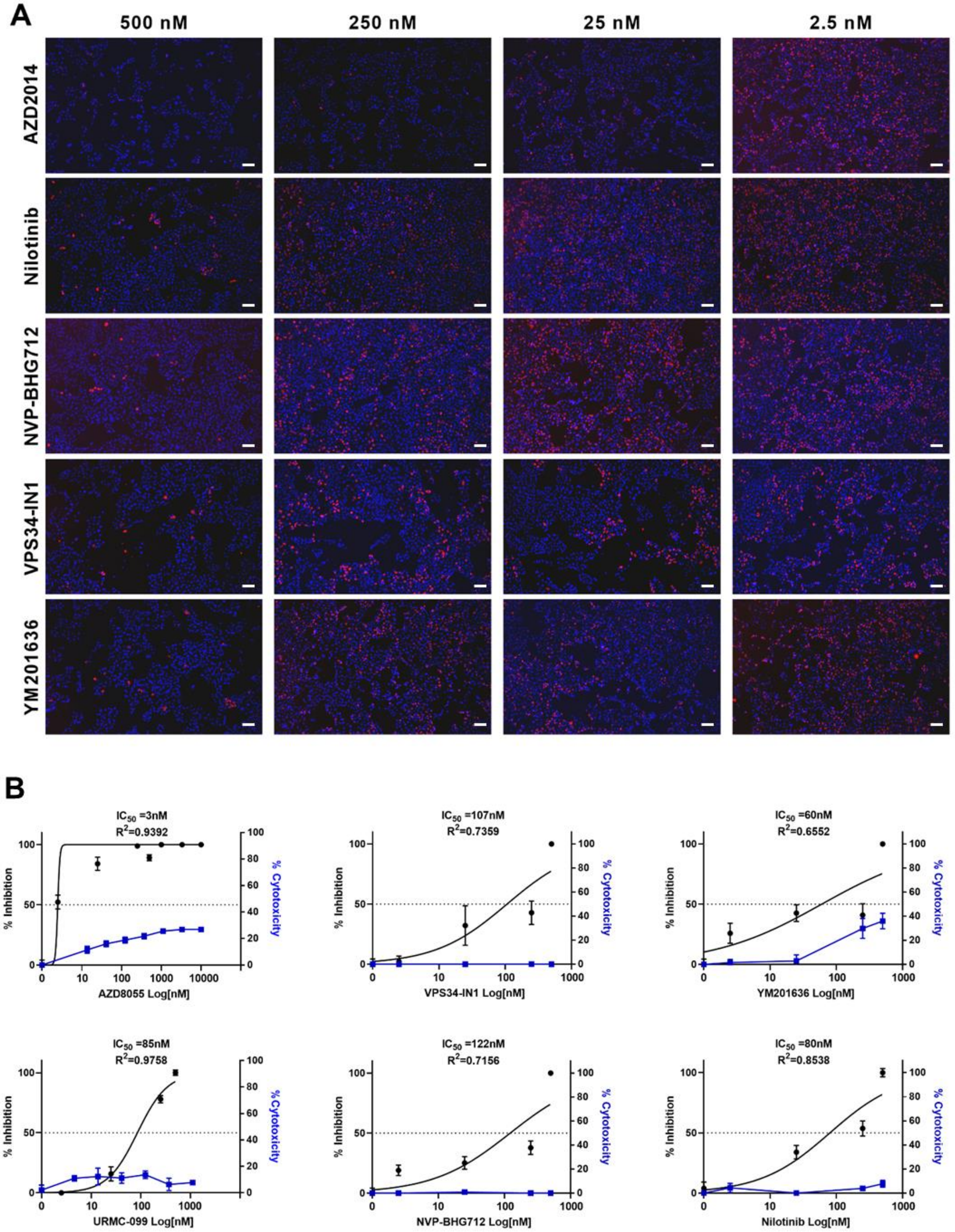
A
500 nM
250 nM
25 nM
2.5 nM
AZD2014
Nilotinib
NVP-BHG712
VPS34-IN1
YM201636
B
IC_{50} =3nM
R^2=0.9392
AZD8055 Log[nM]
IC_{50} =107nM
R^2=0.7359
VPS34-IN1 Log[nM]
IC_{50} =60nM
R^2=0.6552
YM201636 Log[nM]
IC_{50} =85nM
R^2=0.9758
URMC-099 Log[nM]
IC_{50} =122nM
R^2=0.7156
NVP-BHG712 Log[nM]
IC_{50} =80nM
R^2=0.8538
Nilotinib Log[nM]
% Inhibition
% Cytotoxicity

Figure S3. Secondary Screen in Vero E6 cells. (A) Immunofluorescent images of SARS-CoV-2 (red) infected Vero E6 cells treated with indicated drug compounds at various concentrations. (B) Graphs show percent inhibition of SARS-CoV-2 infectivity by indicated compounds. Note: IC50 of each compound is shown in the graph. Related to Figure 1.

Figure S4

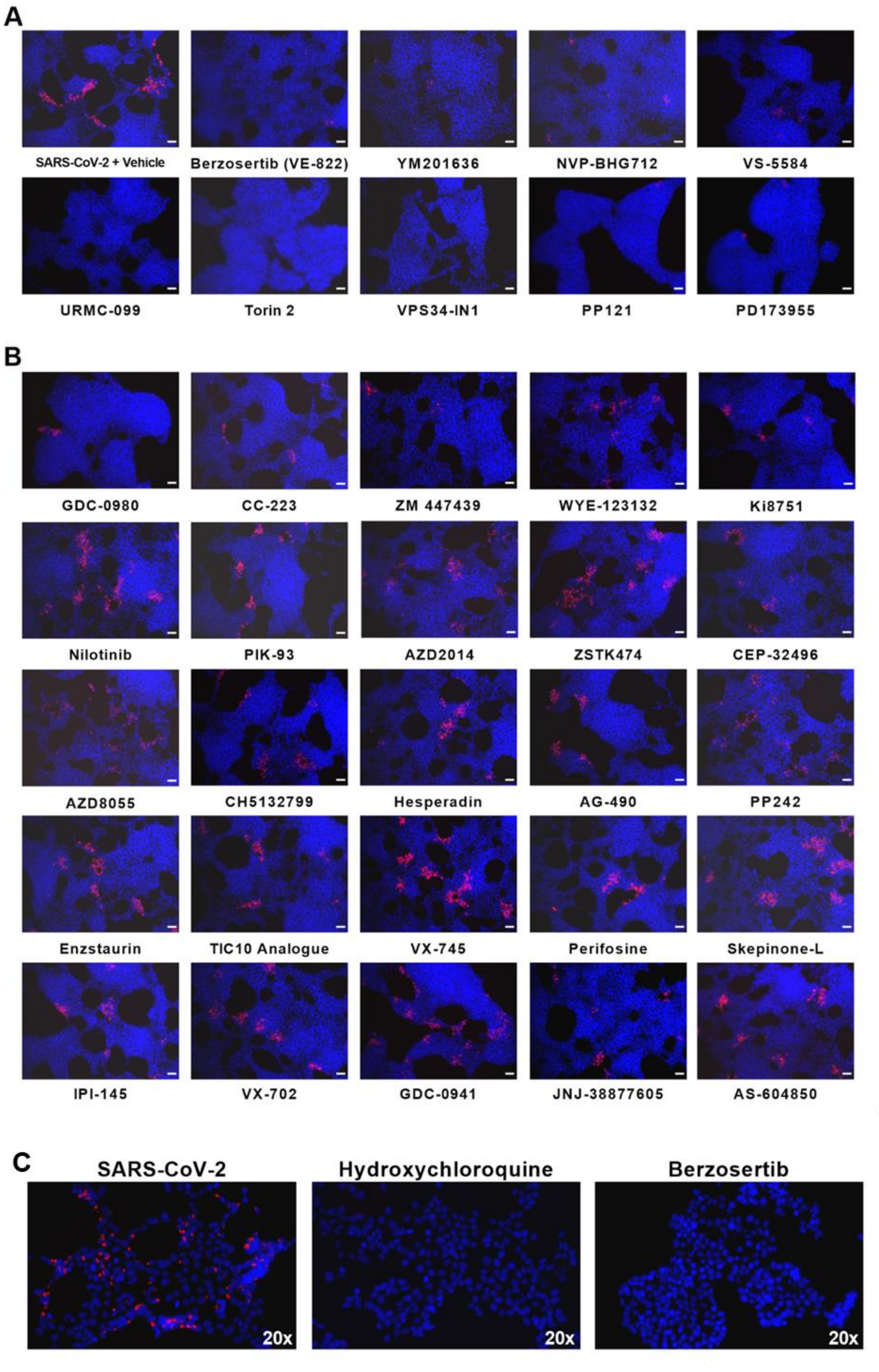
A
SARS-CoV-2 + Vehicle
Berzosertib (VE-822)
YM201636
NVP-BHG712
VS-5584
URMC-099
Torin 2
VPS34-IN1
PP121
PD173955
B
GDC-0980
CC-223
ZM 447439
WYE-123132
Ki8751
Nilotinib
PIK-93
AZD2014
ZSTK474
CEP-32496
AZD8055
CH5132799
Hesperadin
AG-490
PP242
Enzstaurin
TIC10 Analogue
VX-745
Perifosine
Skepinone-L
IPI-145
VX-702
GDC-0941
JNJ-38877605
AS-604850
C
SARS-CoV-2
Hydroxychloroquine
Berzosertib
20x
20x
20x

Figure S4. Secondary Screen in HEK293-ACE2 cells. The cells were pre-treated with each of the 34 compounds (250 nM) from the primary screen for 24 hours. The following day the cells were infected with SARS-CoV-2 with an MOI of 0.1. At one day post-infection the cells were fixed in methanol and immunostained to detect SARS-CoV-2 Spike antigen (red). (A) Immunofluorescent images of compounds having potent anti- SARS-CoV-2 activity are shown. Vehicle control with infection was included as reference. (B) Drugs with low or no anti-viral activity at tested dose of 250 nM are presented. Note: Compounds including nilotinib and AZD2014 had not exhibited potent anti-viral activity at tested 250 nM dose level. Scale Bar=25um. (C) IFA images show SARS-CoV-2 (red) infection in untreated cells and hydroxychloroquine (10 µM) treated cells. Complete inhibition of SARS-CoV-2 infection in berzosertib (100nM) treated cells is noted. 20x magnification. Related to Figure 1.

Figure S5

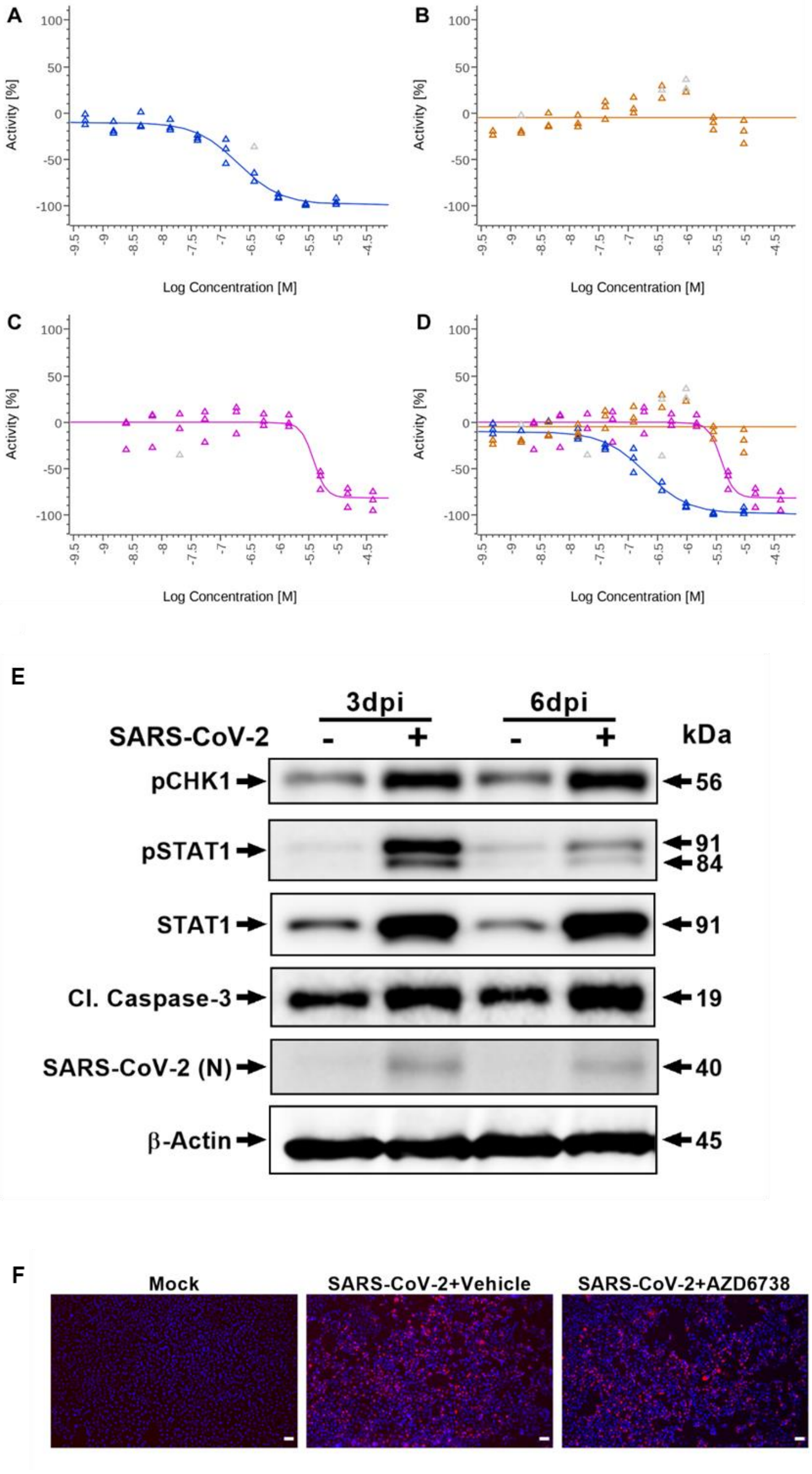
A
B
C
D
Activity [%]
Log Concentration [M]
E
3dpi
6dpi
SARS-CoV-2
kDa
pCHK1
56
pSTAT1
91
84
STAT1
91
Cl. Caspase-3
19
SARS-CoV-2 (N)
40
β-Actin
45
F
Mock
SARS-CoV-2+Vehicle
SARS-CoV-2+AZD6738

Figure S5. Berzosertib inhibits SARS-CoV-2 replication in human HeLa-ACE2 cells and affects DDR pathway. (A) Graph shows antiviral activity measured with a SARS-CoV-2 immunofluorescence signal leading to identification of infected cells with 0% activity equals 100% infected cells. (B) total cells per well in SARS-CoV2 infected cell test with 0% activity equaling no change vs. control (C) total cells per well in HeLa-ACE2 uninfected cell control. (D) Overlay of curves in A, B and C. (E) SARS-CoV-2 activates DDR pathway by CHK1 phosphorylation. Western blot analysis shows phosphorylation of key ATR kinase downstream target protein CHK1 in SARS-CoV-2 infected lung proximal airway epithelial cells grown in air-liquid interface (ALI) culture (48hpi). (F) Evaluating additional DNA-Damage Pathway ATR Kinase Inhibitor. Vero E6 cells pre-treated (24 hours) with AZD6738 (10µM) were infected with SARS-CoV-2 48 hours post-infection virus replication was visualized with immunostaining. Scale Bar=100 µm. IFA images show SARS-CoV-2 (red) infection in vehicle or AZD6738 treated cells. No inhibition of SARS-CoV-2 infection in AZD6738 (10µM) treated cells is noted. Related to Figure 3 and 4.

SUPPLEMENTARY TABLE

Table S2. Drugs compounds selected from primary screen having antiviral activity at 250 nM concentration (Related to Figure 1).

	**Compound Name**	**Activity**
1	Berzosertib (M6620)	ATR kinase inhibitor
2	Nilotinib (AMN-107)	Bcr-Abl inhibitor
3	NVP-BHG712	EphB4 inhibitor
4	VPS34-IN1	Vps34 inhibitor
5	YM201636	PIKfyve inhibitor
6	AZD-2014 (Vistusertib)	Inhibitor mTOR and multiple PI3K isoforms (α/β/γ/δ)
7	AZD8055	ATP-competitive mTOR inhibitor
8	VS-5584 (SB2343)	Dual PI3K/mTOR inhibitor
9	Torin 2	Selective mTOR inhibitor
10	CC-223 (Onatasertib)	mTOR inhibitor
11	WYE-125132 (WYE-132)	ATP-competitive mTOR inhibitor
12	PP242 (Torkinib)	mTOR inhibitor
13	ZSTK474	Inhibitor of class I PI3K isoforms
14	GDC-0941 (Pictilisib)	PI3Kα/δ inhibitor
15	GDC-0980 (RG7422)	Class I PI3K inhibitor for PI3Kα/β/δ/γ
16	AS-604850	ATP-competitive PI3Kγ inhibitor
17	CH5132799	Inhibitor of class I PI3Ks
18	IPI-145 (INK1197)	selective PI3K δ/γ inhibitor
19	PIK-93	PI3Kγ and PI4KIIIβ inhibitor
20	Enzastaurin (LY317615)	PKCβ inhibitor
21	TIC10 Analogue	Inactivates Akt and ERK
22	Perifosine (KRX-0401)	Akt inhibitor
23	AG-490 (Tyrphostin B42)	EGFR and JAK2 inhibitor
24	VX-745	p38α MAPK inhibitor
25	Skepinone-L	p38α-MAPK inhibitor
26	VX-702	p38α MAPK inhibitor
27	CEP-32496	Inhibitor of BRAF(V600E/WT) and c-Raf
28	ZM 447439	ATP-competitive inhibitor for Aurora kinases A and B
29	Hesperadin	Aurora kinase B inhibitor
30	JNJ-38877605	ATP-competitive inhibitor of c-Met
31	Ki8751	VEGFR2 inhibitor
32	URMC-099	Mixed lineage kinase (MLK) inhibitor
33	PD173955	Bcr-Abl inhibitor
34	PP121	Inhibitor of PDGFR, Hck, mTOR, VEGFR2, Src and Abl
