# Supplementary material for: Antiviral drug screen identifies DNA-damage response inhibitor as potent blocker of SARS-CoV-2 replication
Source: Cell Rep. 2021 Mar 18;35(1):108940. doi: 10.1016/j.celrep.2021.108940 (PMC7969873; doi:10.1016/j.celrep.2021.108940)
Supplement: Document S2. Article plus supplemental information [file mmc5.pdf]

Cell Reports

Report

Antiviral drug screen identifies DNA-damage response inhibitor as potent blocker of SARS-CoV-2 replication

Graphical abstract

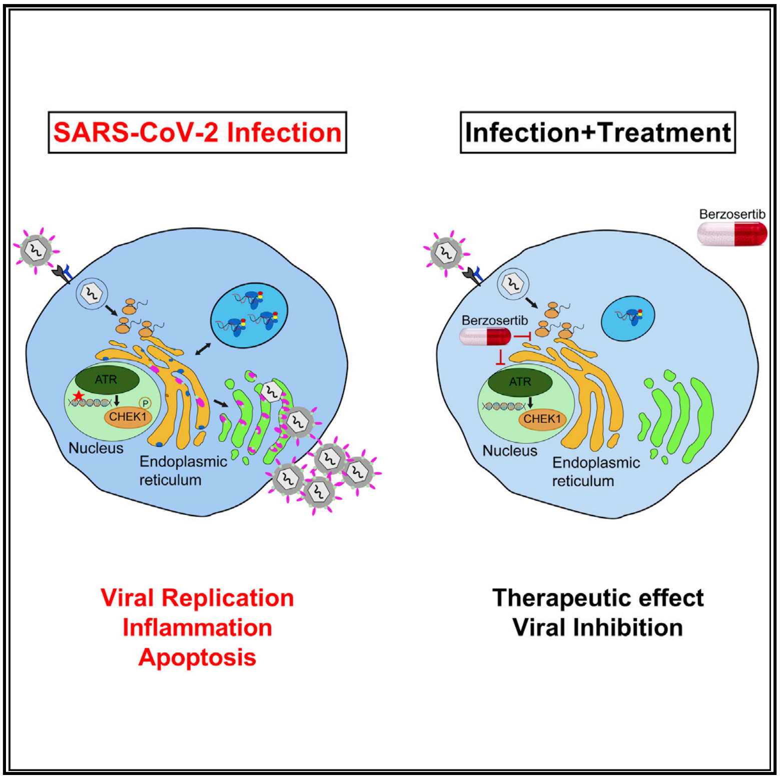

Highlights

- Kinase inhibitor screen identified 34 compounds with anti-SARS-CoV-2 activity
- Inhibitors targeted mTOR-PI3K-AKT and DNA-damage response (DDR) signaling pathways
- ATR kinase inhibitor berzosertib blocked SARS-CoV-1, SARS-CoV-2, and MERS-CoV infection
- Treatment with berzosertib blocks SARS-CoV-2 at post-entry level in epithelial cells

Authors

Gustavo Garcia, Jr., Arun Sharma, Arunachalam Ramaiah, ..., Ulrich A.K. Betz, Robert D. Damoiseaux, Vaithilingaraja Arumugaswami

Correspondence

ulrich.betz@merckgroup.com (U.A.K.B.), rdamoiseaux@mednet.ucla.edu (R.D.D.), varumugaswami@mednet.ucla.edu (V.A.)

In brief

Garcia et al. screen a library of drug compounds and identify SARS-CoV-2-specific antiviral agents. These drugs have been shown to modulate cellular signaling cascades, including mTOR-PI3K-AKT and DNA-damage response (DDR) pathways. A highly effective drug candidate, berzosertib, blocked multiple coronaviruses, such as SARS-CoV-1, SARS-CoV-2, and MERS-CoV, thus providing a potential therapeutic against COVID-19.

Garcia et al., 2021, Cell Reports *35*, 108940
April 6, 2021 © 2021 The Author(s).
https://doi.org/10.1016/j.celrep.2021.108940

Cell Reports

CellPress
OPEN ACCESS

Report

Antiviral drug screen identifies DNA-damage response inhibitor as potent blocker of SARS-CoV-2 replication

Gustavo Garcia, Jr.,[1] Arun Sharma,[2,3] Arunachalam Ramaiah,[4,5] Chandani Sen,[6] Arunima Purkayastha,[6] Donald B. Kohn,[6,7,8] Mark S. Parcells,[9] Sebastian Beck,[10] Heeyoung Kim,[11] Malina A. Bakowski,[12] Melanie G. Kirkpatrick,[12] Laura Riva,[12] Karen C. Wolff,[12] Brandon Han,[13] Constance Yuen,[13] David Ulmert,[1,7] Prabhat K. Purbey,[14] Phillip Scumpia,[14] Nathan Beutler,[15] Thomas F. Rogers,[15,16] Arnab K. Chatterjee,[12] Gülsah Gabriel,[10] Ralf Bartenschlager,[11,17,18] Brigitte Gomperts,[6,7,8] Clive N. Svendsen,[2] Ulrich A.K. Betz,[19,*] Robert D. Damoiseaux,[1,7,13,20,*] and Vaithilingaraja Arumugaswami[1,8,13,21,*]

[1]Department of Molecular and Medical Pharmacology, University of California, Los Angeles, Los Angeles, CA 90095, USA
[2]Board of Governors Regenerative Medicine Institute, Cedars-Sinai Medical Center, Los Angeles, CA 90048, USA
[3]Smidt Heart Institute, Cedars-Sinai Medical Center, Los Angeles, CA 90048, USA
[4]Department of Ecology and Evolutionary Biology, University of California, Irvine, Irvine, CA 92697, USA
[5]Section of Cell and Developmental Biology, University of California, San Diego, San Diego, CA 92093, USA
[6]UCLA Children's Discovery and Innovation Institute, Mattel Children's Hospital UCLA, Department of Pediatrics, David Geffen School of Medicine, UCLA, Los Angeles, CA 90095, USA
[7]Jonsson Comprehensive Cancer Center, UCLA, Los Angeles, CA 90095, USA
[8]Eli and Edythe Broad Center of Regenerative Medicine and Stem Cell Research, UCLA, Los Angeles, CA 90095, USA
[9]Department of Animal and Food Sciences, Department of Biological Sciences, University of Delaware, Newark, DE 19716, USA
[10]Heinrich Pette Institute, Leibniz Institute for Experimental Virology, Hamburg, Germany
[11]Department of Infectious Diseases, Molecular Virology, Heidelberg University, Heidelberg, Germany
[12]Calibr, a division of Scripps Research Institute, 11119 North Torrey Pines Road, La Jolla, CA 92037, USA
[13]California NanoSystems Institute, University of California, Los Angeles, Los Angeles, CA 90095, USA
[14]Department of Pathology and Laboratory Medicine, University of California, Los Angeles, Los Angeles, CA 90095, USA
[15]Department of Immunology and Microbiology, Scripps Research Institute, 10550 North Torrey Pines Road, La Jolla, CA 92037, USA
[16]UC San Diego Division of Infectious Diseases and Global Public Health, UC San Diego School of Medicine, La Jolla, CA 92093, USA
[17]German Center for Infection Research, Heidelberg partner site, Heidelberg, Germany
[18]Division Virus-Associated Carcinogenesis, German Cancer Research Center (DKFZ), Heidelberg, Germany
[19]Merck KGaA, Darmstadt, Germany
[20]Department of Bioengineering, University of California, Los Angeles, Los Angeles, CA 90095, USA
[21]Lead contact
*Correspondence: ulrich.betz@merckgroup.com (U.A.K.B.), rdamoiseaux@mednet.ucla.edu (R.D.D.), varumugaswami@mednet.ucla.edu (V.A.)
https://doi.org/10.1016/j.celrep.2021.108940

SUMMARY

SARS-CoV-2 has currently precipitated the COVID-19 global health crisis. We developed a medium-throughput drug-screening system and identified a small-molecule library of 34 of 430 protein kinase inhibitors that were capable of inhibiting the SARS-CoV-2 cytopathic effect in human epithelial cells. These drug inhibitors are in various stages of clinical trials. We detected key proteins involved in cellular signaling pathways mTOR-PI3K-AKT, ABL-BCR/MAPK, and DNA-damage response that are critical for SARS-CoV-2 infection. A drug-protein interaction-based secondary screen confirmed compounds, such as the ATR kinase inhibitor berzosertib and torin2 with anti-SARS-CoV-2 activity. Berzosertib exhibited potent antiviral activity against SARS-CoV-2 in multiple cell types and blocked replication at the post-entry step. Berzosertib inhibited replication of SARS-CoV-1 and the Middle East respiratory syndrome coronavirus (MERS-CoV) as well. Our study highlights key promising kinase inhibitors to constrain coronavirus replication as a host-directed therapy in the treatment of COVID-19 and beyond as well as provides an important mechanism of host-pathogen interactions.

INTRODUCTION

The current pandemic is caused by a newly discovered coronavirus, severe-acute-respiratory-syndrome-related coronavirus 2 (SARS-CoV-2). Currently, the disease has spread to 215 countries or territories, and the number of coronavirus disease 2019 (COVID-19) cases has surpassed 99 million globally, with more than two million deaths (Dong et al., 2020; WORLDOMETER, 2020). SARS-CoV-2 is a zoonotic virus having similarity with bat SARS-CoV-like viruses (Ramaiah and Arumugaswami, 2020).

This is an open access article under the CC BY license (http://creativecommons.org/licenses/by/4.0/).

COVID-19 is a multi-organ disease affecting lung, heart, kidney, and brain (Hou et al., 2020; Xu et al., 2020b, Dolhnikoff et al., 2020; Fanelli et al., 2020; Puelles et al., 2020; Lu et al., 2020; Paterson et al., 2020). This virus enters into a host cell by binding its transmembrane spike glycoprotein (S protein) to the cellular membrane angiotensin-converting enzyme 2 (ACE2) receptor, which is expressed in various organs (Kai and Kai, 2020). A gradient of ACE2 expression has been found in the respiratory tract with the highest levels in the nose and decreasing expression in the lower respiratory tract (Hou et al., 2020). In the proximal airway, SARS-CoV-2 infected all cell types, whereas type-2 alveolar cells (AT2) were found to be infected in the distal airway (Hou et al., 2020). ACE2 is also expressed in other organs, such as the kidney, heart, and intestines (Li et al., 2020; Zou et al., 2020; Xu et al., 2020a). The major cause of morbidity and mortality from COVID-19 is acute lung injury with diffuse alveolar damage resulting in acute respiratory distress syndrome (ARDS) (Xu et al., 2020b). Moreover, there have been reports of patients exhibiting acute kidney injury (Pacciarini et al., 2008; Fanelli et al., 2020; Puelles et al., 2020), vascular inflammation (endotheliitis), and cardiac complications (Varga et al., 2020; Yancy and Fonarow, 2020; Dolhnikoff et al., 2020; Grimaud et al., 2020; Belhadjer et al., 2020; Sanna et al., 2020; Escher et al., 2020; Tavazzi et al., 2020; Gnecchi et al., 2020; Craver et al., 2020). Underlying cardiac ailments, diabetes, and obesity are linked to increased risk of mortality (Shi et al., 2020; Fried et al., 2020). This results from viral replication in epithelial cells causing cell injury, a vigorous inflammation-dominated response, organ failure, and possibly, death.

Developing a therapeutic that prevents viral replication is likely to significantly reduce the severity of COVID-19 disease in affected individuals. RNA viruses mutate during each round of genome replication because of the error-prone nature of viral RNA-dependent RNA polymerase (RdRp) and, therefore, develop resistance to direct-acting antivirals agents (DAAs). Viruses take over many host kinases at distinct steps of their life cycle (Supekova et al., 2008; Li et al., 2009; Keating and Striker, 2012; Jiang et al., 2014); thus, the kinases represent attractive targets for broad-spectrum therapy. These findings, combined with the development and approval of many kinase inhibitors for the treatment of cancer (Gross et al., 2015) and inflammatory conditions (Ott and Adams, 2011) have sparked efforts aimed to determine the therapeutic potential of such drugs to combat viral infections. The high average cost (more than two billion dollars) and long timeline (8–12 years) to develop a new drug (Tufts Center for the Study of Drug Development, 2014), limit the scalability of the DAA approach to drug development, particularly with respect to emerging viruses. This approach is, therefore, not feasible for the short-term development of a cure that is specific for SARS-CoV-2. The screening of approved drugs to identify therapeutics for drug repurposing is an effective approach that has been used for many viral diseases, including SARS-CoV-2 (Schor and Einav, 2018; García et al., 2012; Johansen et al., 2013; Madrid et al., 2013; Riva et al., 2020; Jeon et al., 2020; Weston et al., 2020; Dyall et al., 2014; Coleman et al., 2016; Weisberg et al., 2020). Thus, our strategy for developing COVID-19 treatment is based on two main facts about the disease: (1) all patients presenting with symptoms have been infected with SARS-CoV-2, and the virus has gained entry into the airway cells; and (2) viruses are dependent on cellular proteins for each step of their life cycle, and they hijack many host cell factors for their replication. Moreover, these host proteins are not subject to evolutionary pressure because of the short duration of acute infection; therefore, there is limited chance of emergence of drug-resistant viral mutants.

RESULTS AND DISCUSSION

To shed light on an effective antiviral therapy for COVID-19 treatment, we established a SARS-CoV-2 infectious cell culture system and virological assays using Vero E6 cells. The SARS-CoV-2 isolate USA-WA1/2020 was obtained from BEI Resources of the National Institute of Allergy and Infectious Diseases (NIAID), and studies involving live virus were conducted in a biosafety level 3 (BSL3) high-containment facility. The SARS-CoV-2 was passaged once in Vero E6 cells, and viral stocks were aliquoted and stored at −80°C. Virus titer was measured in Vero E6 cells by median tissue culture infection dose ($TCID_{50}$) assay. Striking cytopathic effect (CPE) was observed in SARS-CoV-2-infected cells (Figure S1A), indicating viral replication and associated cell injury. At 48 h post infection (hpi), viral infection was examined by immunofluorescence assay (IFA) analysis using SARS-CoV spike (S) antibody. Spike protein was detected in the cytoplasm of the infected cells, revealing the presence of viral infection (Figure S1B). We also demonstrated that the drugs hydroxychloroquine (HQ; 10 μM), a known endosomal acidification inhibitor, as well as interferon (IFN)-β, IFN-α, and EIDD-2801 (molnupiravir), effectively blocked SARS-CoV-2 infection (Liu et al., 2020) (Figure S1C). Therefore, we used this platform for subsequent drug-screening studies.

To evaluate the antiviral properties of cellular protein kinase inhibitors, we performed medium-throughput primary drug screening (Figure 1A) by selecting a drug compound library that broadly covers 430 kinase inhibitors with the screening compound concentration that would have potent anti-viral activity with low levels of toxicity. There are only 518 human protein kinases described, with 478 kinases belonging to a single superfamily. The limitation in the number of druggable kinases, along with our criterion of selecting kinase inhibitors that are being evaluated for clinical studies, led to the use of 430 compounds. These kinase inhibitors have typically been tested for oncologic and immunologic indications, for which we have clinical trial phase 1, 2, and 3 data (Table S1), but no data available on SARS-CoV-2. Because this kinase inhibitor library targets cancer indications, we decided to avoid using human lung cancer epithelial cell lines. Drug compounds were formulated into DMSO and pre-plated into media at a 2× concentration (final drug concentration, 250 nM). Compounds were added to the Vero E6 cells in the BSL3 laboratory, followed by SARS-CoV-2 at a multiplicity of infection (MOI) of 0.1. After the 48-h incubation at 37°C, 5% CO_2, viral CPE was scored and imaged (Figure 1A). The compounds that prevented the viral CPE were identified (Table S2; Figure S2) and subjected to pathway analysis (Figures 1B and 1C). The drug-cellular-protein-interaction network was created by mining the collection of 34 hit compounds against the STITCH (search tool for interactions of

CellPress
OPEN ACCESS

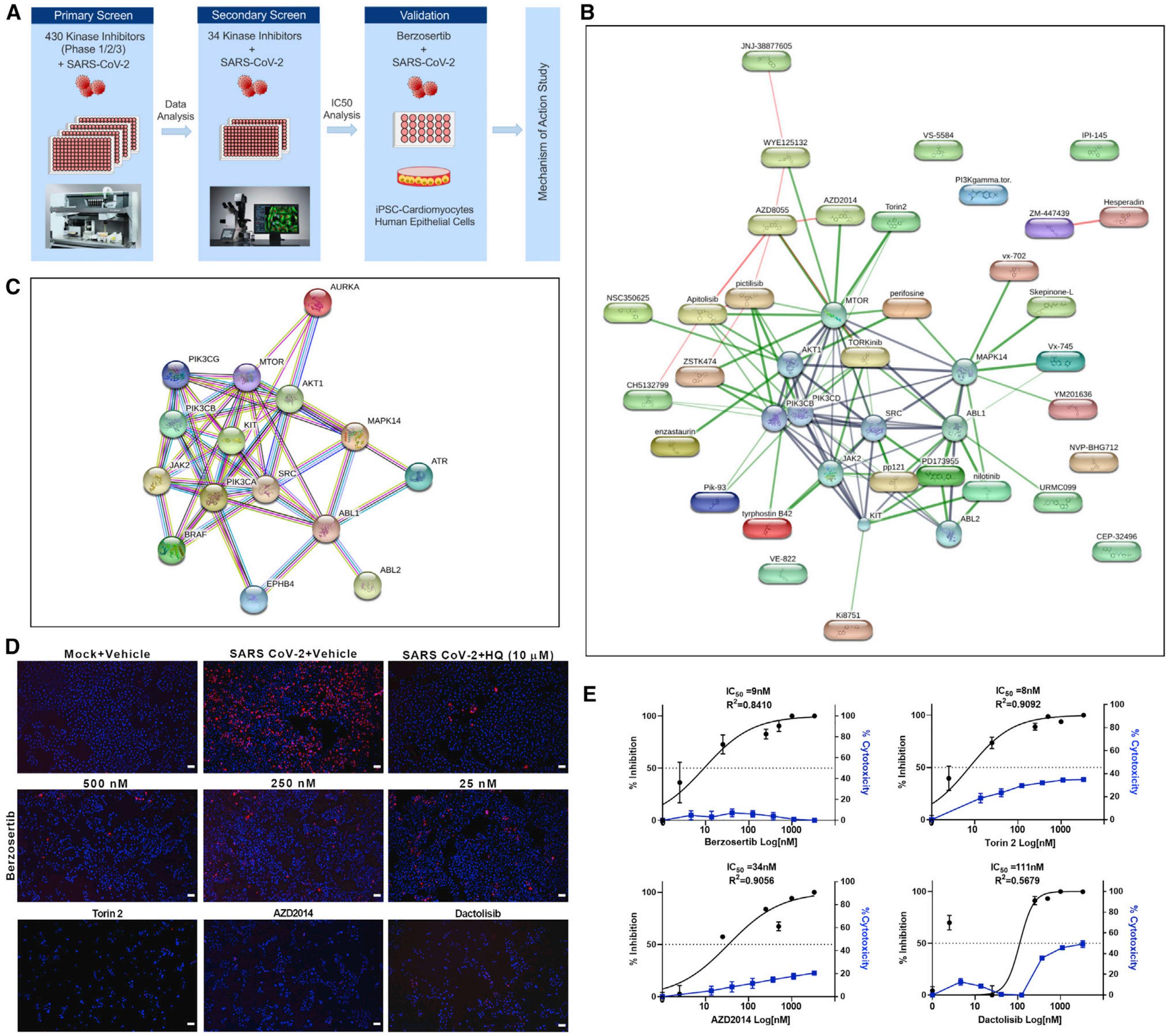

Figure 1. Drug-target kinase-connectivity network identifies key anti-viral protein kinase inhibitors

(A) Workflow of drug screen is shown.

(B) Connectivity map of drug hits from the primary screen is illustrated. The graphical representation shown is the confidence view in which stronger associations are represented by thicker lines, protein-protein interactions are shown in gray, chemical-protein interactions are in green, and interactions between chemicals are in red. Round shapes represent proteins, and oval shapes indicate hit compounds from the primary screen. The analysis indicated a protein-protein interaction enrichment score of 0.0026, which is statistically significant.

(C) STRING analysis of host protein-protein network identified from the drug screen is shown.

(D) Immunofluorescent images of SARS-CoV-2-infected cells (red) treated with the indicated drug compounds at various concentrations. AZD2014, torin2, and dactolisib were used at 500 nM. Scale bar, 100 μm.

(E) Graphs show the percentage of inhibition of SARS-CoV-2 infectivity and cytotoxicity by the indicated compounds. Note: IC_{50} of each compound is shown in the graph. Representative data from two independent experiments are presented.

chemicals) database, in an unbiased fashion, using the standard and unmodified settings (Kuhn et al., 2008). The compounds onatasertib and VPS34-IN1 are not in the STITCH database and were, thus, excluded from the network map analysis. The hit compounds targeted a few selected kinases, such as mTOR, AKT, PI3K, SRC, ABL, and ATR, and a limited set of pathways, such as mTOR-PI3K-AKT, ABL-BCR/MAPK, and DNA-damage response (DDR) (Figures 1B and 1C), suggesting the specific nature of the identified antiviral agents.

To rapidly confirm and prioritize the most promising compounds according to their anti-SARS-CoV-2 activity, we selected 34 compounds from the primary screening for secondary screening with

multiple drug doses (2.5, 25, 250, and 500 nM) in triplicate, using 96-well plates. We used an IFA to quantify viral-infected cells from each well. This comprehensive screen in Vero E6 cells, as well as HEK293-ACE2 cells, verified many hits of the primary screen (Figures 1B–1D, S3, and S4). In Vero E6 cells, compounds berzosertib (M6620), torin2, and vistusertib (AZD2014) all demonstrated antiviral activity at half-maximal inhibitory concentration (IC_{50}) less than 25 nM, whereas nilotinib, NVP-BHG712, VPS34-INI, URMC-099, and YM201636 showed IC_{50} ranges between 50 and 125 nM (Figures 1E and 3). We also included an additional kinase inhibitor, dactolisib, in the secondary validation step. These compounds act against SARS-CoV-2 by limiting viral infection through inhibiting critical cellular enzymes needed for viral replication. In contrast, nucleoside analogs, such as remdesivir, EIDD-2801 (molnupiravir), and ribavirin have been shown to inhibit the viral RdRp enzyme and could also induce compromising errors during viral genome replication. Our secondary screen confirmed the antiviral activities of these kinase inhibitors and the critical cellular pathways subjected to activation, suppression, or some form of modulation by viral infection. Interestingly, we observed that several antiviral compounds targeted the mTOR-PI3K-AKT pathway, including dactolisib, AZD2014, and torin2 (Figure 1B). In general, we observed that compounds blocking this pathway had relatively increased cytotoxicity. The mammalian target of rapamycin (mTOR) regulates cell growth, autophagy, and various metabolic processes (Le Sage et al., 2016; Weichhart, 2012). The mTOR-PI3K-AKT pathway has been shown to be targeted by various viruses, including influenza virus, herpesvirus, hepatitis C virus, and adenovirus (Sodhi et al., 2006; Bose et al., 2012; Le Sage et al., 2016; Kong et al., 2014; Moody et al., 2005). The torin compound has been shown to inhibit virus replication by blocking mTOR kinase (Kuss-Duerkop et al., 2017). Dactolisib has also shown to inhibit HIV-1 replication (Campbell et al., 2018). Our findings suggest that dactolisib, AZD2014, and torin2, targeting the PI3K/AKT1/MTOR pathway, could be developed as potential therapeutics against COVID-19.

To further confirm drug efficacy and to investigate mechanism of action, we tested drugs using human cells. We focused on a class of antiviral compound from our screen, berzosertib, which is a DDR pathway inhibitor targeting the protein kinase ATR (ataxia telangiectasia and Rad3-related protein) and is already in phase 2 clinical trials for solid tumors (Konstantinopoulos et al., 2020; Yap et al., 2020). First, we used an ACE2 entry receptor overexpressing HEK293-ACE2 cells for infection. Analysis of SARS-CoV-2-infected cells treated with berzosertib (100 nM) showed complete inhibition of virus replication at 48 hpi (Figure S4C). This result provides additional confirmation of berzosertib as an efficient candidate for the treatment of SARS-CoV-2 infection.

Emerging evidence indicates that the heart is directly affected by SARS-CoV-2 (Varga et al., 2020; Yancy and Fonarow, 2020; Dolhnikoff et al., 2020; Grimaud et al., 2020; Belhadjer et al., 2020; Sanna et al., 2020; Escher et al., 2020; Tavazzi et al., 2020; Gnecchi et al., 2020; Craver et al., 2020). Thus, we used the human-induced pluripotent stem-cell-derived cardiomyocyte (hiPSC-CM) system for antiviral drug testing against SARS-CoV-2. Recent studies have reported that hiPSC-CMs are more efficient at recapitulating cardiovascular diseases at a cellular level (Lan et al., 2013; Sun et al., 2012) and have demonstrated susceptibility to SARS-CoV-2 infection (Sharma et al., 2020). Thus, this cardiomyocyte system is useful for antiviral drug testing against SARS-CoV-2. To evaluate potency, hiPSC-CMs were infected with SARS-CoV-2 and treated with berzosertib (250 nM). Untreated SARS-CoV-2-infected cells had significantly reduced cardiomyocyte beating with non-synchronous twitching of a few clusters of cells (refer to Videos S1 and S2). We found treatment with berzosertib stabilized cardiomyocyte function and showed similar beats per minute to uninfected cardiomyocytes (Figure 2A; Videos S1 and S2). Cardiomyocytes were also treated with dactolisib because of its potent antiviral activity to target the mTOR-PI3K-AKT pathway. Although dactolisib inhibited viral production, it affected the cardiomyocyte functional beating, suggesting cardiotoxicity at the tested dose. The inhibition of virus production by berzosertib was evaluated by quantifying the infectious virus (TCID50) in the supernatant at various time points (Figure 2B). We observed berzosertib treatment significantly reduced virus production as well as apoptotic cell death associated with viral infection (Figures 2C and 2D). Infection of cardiomyocytes with SARS-CoV-2 was confirmed by specific staining of cardiac troponin T (cTnT) and viral spike proteins (Figure 2E). Untreated SARS-CoV-2-infected cells had infection-mediated cell injury with disrupted troponin T fibers. No *in vitro* cardiotoxicity was associated with berzosertib at the tested dose (250 nM), whereas dactolisib had cardiotoxicity at the same dose.

Antiviral activity of berzosertib was independently found in an antiviral screen conducted with a HeLa-ACE2/SARS-CoV-2 infection assay combined with an uninfected HeLa-ACE2 counter-screen (Figures S5A–S5D). Berzosertib exhibited a median effective dose (ED_{50}) = 0.2 μM measured as the percentage of infected cells. The compound did not show cytotoxic effect at its active concentrations, i.e., it did not change the total cells per well (Figure S5), as evidenced via a median cytotoxic concentration (CC_{50}) = 3.89 μM in an uninfected HeLa-ACE2 counter-screen. In the same assay conditions, remdesivir resulted in an ED_{50} = 0.124 μM and a CC_{50} > 10 μM.

Next, berzosertib was tested for antiviral activity against SARS-CoV-2, SARS-CoV-1, and Middle Eastern respiratory syndrome coronavirus (MERS-CoV) on human airway epithelial cells, Calu-3 (Figure 3). Calu-3 cells were infected, treated with berzosertib, and at 48 hpi, supernatants were collected and titrated on Vero E6 cells to determine viral titers and IC_{50} values. Berzosertib exhibited an IC_{50} = 0.48 μM for SARS-CoV2 (Figure 3A) with a similar activity against SARS-CoV1 (Figure 3C) and MERS-CoV (Figure 3D). In comparison, remdesivir under the same assay conditions showed an IC_{50} = 0.15 μM (Figure 3B). In an assay conducted with A549-ACE2 cells infected with SARS-CoV-2, berzosertib exhibited an IC_{50} = 0.22 ± 0.03 μM; selectivity index (SI) = 204. Interestingly, it could be demonstrated that berzosertib is acting in a synergistic manner in combination treatment with remdesivir, which showed an IC_{50} of 0.2 μM in the same system (Figure 3F). The respective inhibition curves and the isobologram are shown in Figures 3E–3G. Isobologram analysis was performed with the Compusyn software package (Chou, 2006). The isobologram indicates synergistic antiviral activity between remdesivir and berzosertib (Figure 3H). This observation is interesting because remdesivir blocks

CellPress
OPEN ACCESS

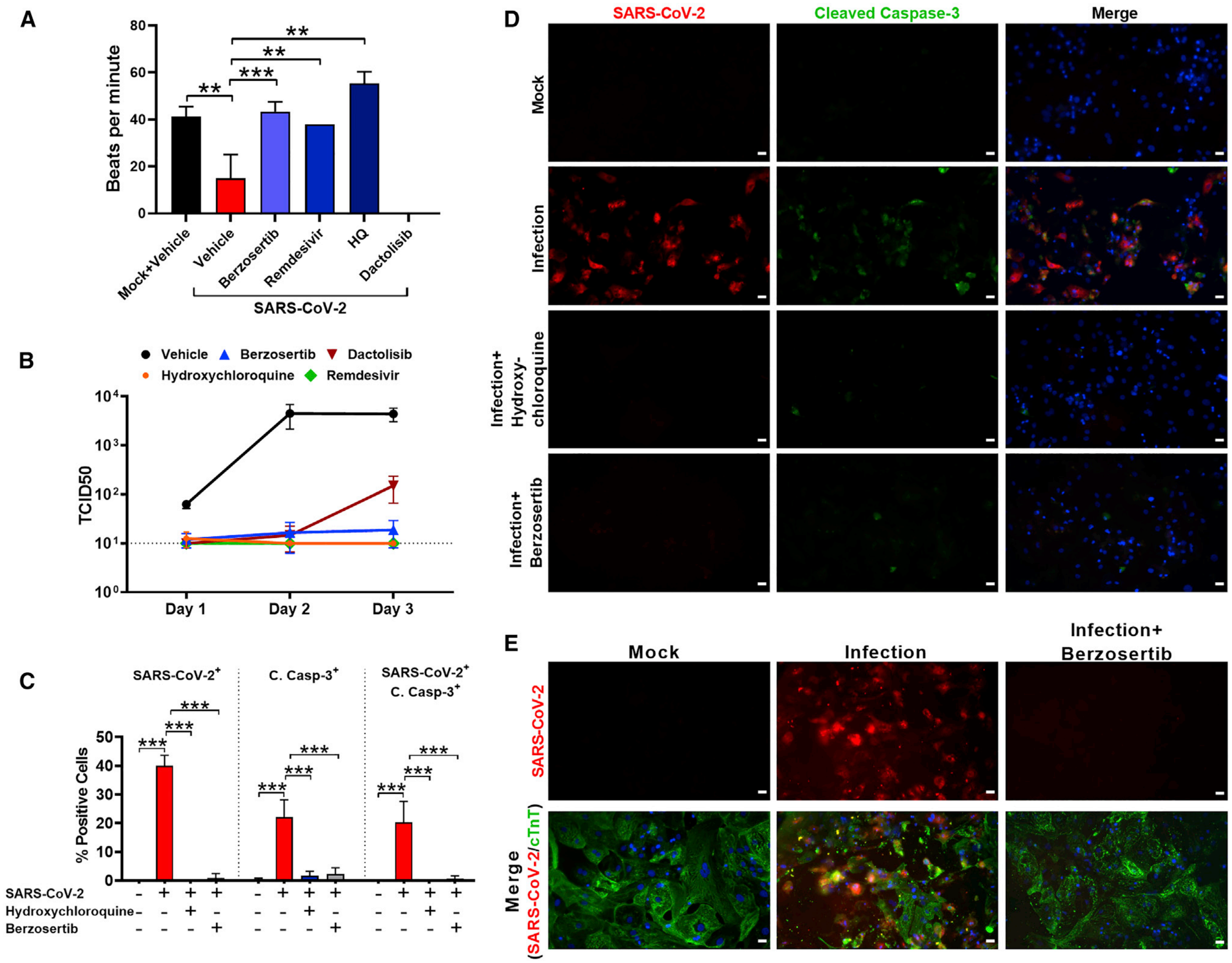

Figure 2. Berzosertib inhibits SARS-CoV-2 replication in hiPSC-CMs

(A) Graph shows beats per minute of SARS-CoV-2-infected hiPSC-CM cells treated with berzosertib (250 nM), dactolisib (250 nM), remdesivir (10 μM), and HQ (10 μM).

(B) Graph shows viral titer ($TCID_{50}$/mL) of supernatant collected at the indicated time points after SARS-CoV-2 infection of drug-treated hiPSC-CMs.

(C) Graph depicts quantification of SARS-CoV-2 and cleaved caspase-3-positive cells.

(D) IFA images of hiPSC-CMs undergoing apoptosis after SARS-CoV-2 infection and drug treatment at 72 hpi. Scale bar, 25 μm.

(E) hiPSC-CMs were stained with cardiac troponin T (cTnT) (green) to demonstrate that cells are protected from SARS-CoV-2-mediated cell injury (red) by berzosertib (250 nM). Scale bar, 25 μm.

Statistical analysis of graphs (A and C) was conducted by multiple-comparison one-way analysis of variance (ANOVA) was conducted. **$p < 0.001$, ***$p < 0.0001$. Representative data from three independent experiments are presented.

SARS-CoV-2 genomic RNA replication by inhibiting viral RNA polymerase, which, in turn, can rule out RNA polymerase as a target of berzosertib. Thus, the observed synergy can be due to the effects on independent targets.

Lastly, we tested berzosertib in a primary human lung tissue culture system consisting of mucociliary air-liquid interface (ALI) cultures derived from primary human tissue (Purkayastha et al., 2020). In this ALI system, as well, berzosertib was effective in inhibiting SARS-CoV-2 (Figures 4A and 4B). Taken together, our results on a human primary cell system suggest that berzosertib is a potent and safe class of antivirals against coronavirus infections with a low risk of cardiac adverse events.

We further investigated a potential mode of action of berzosertib in modulating host cellular signaling pathways. Berzosertib is a selective inhibitor of serine/threonine-protein kinase ATR, which is an important member of the DDR pathway. Berzosertib has been previously shown to block the ATR-CHK1 pathway in cancer cells, with no discernable effect on normal cells (Mei et al., 2019). In healthy cells, the ATR-CHK1 pathway is required for maintaining cellular genome integrity. ATR kinase also has an important role as a replication stress sensor that can switch off cell proliferation if DNA damage is sensed. Studies have shown that berzosertib blocks the phosphorylation of downstream signaling factor

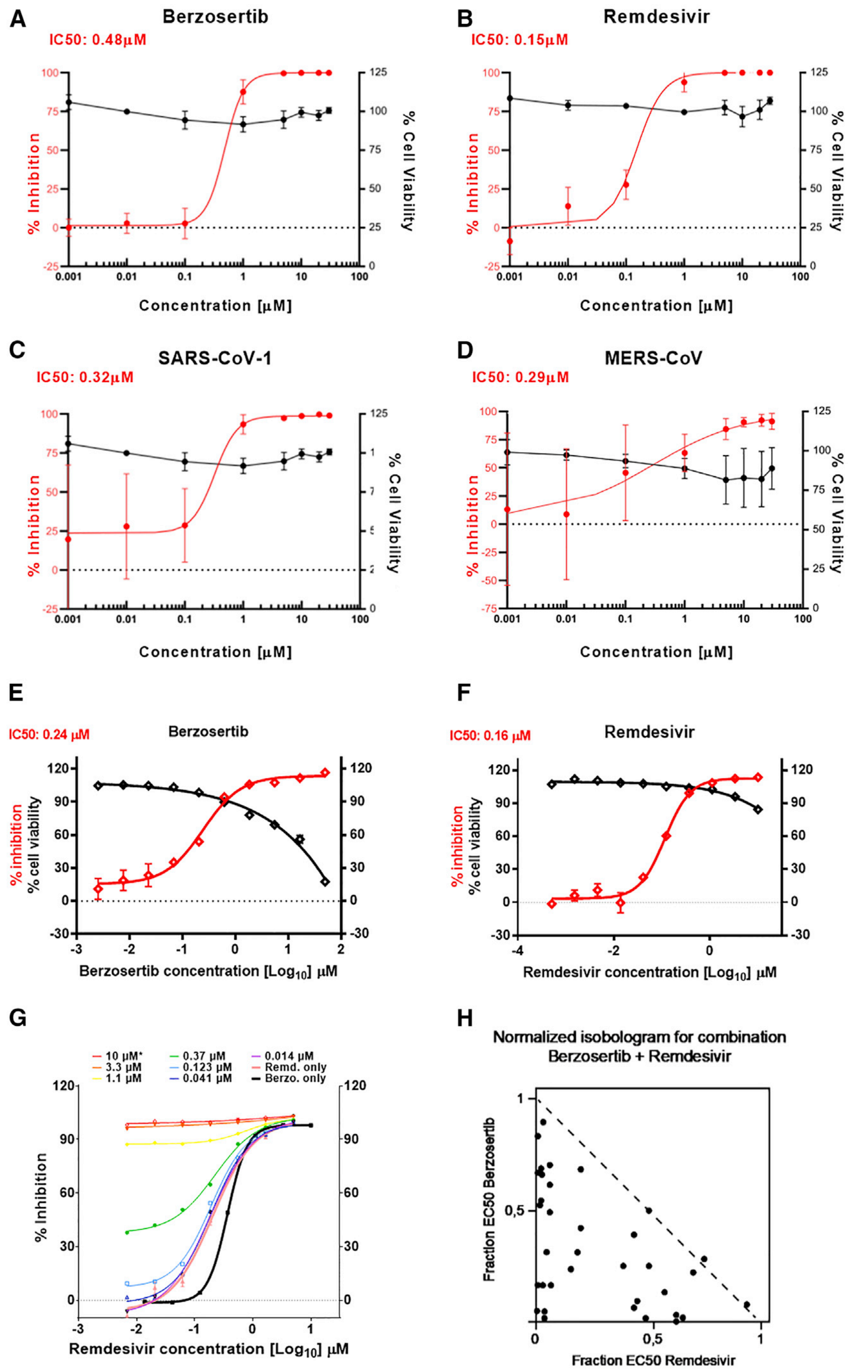

Figure 3. Berzosertib inhibits SARS-CoV-2, SARS-CoV-1, and MERS-CoV replication in human cells and is synergistic with remdesivir

(A and B) Graphs show an eight-point dose-response curve of berzosertib (A) or remdesivir (B) in SARS-CoV-2-infected Calu-3 cells. Contrasted with cell viability of mock-infected cells.

(C and D) Antiviral effect of berzosertib on SARS-CoV-1 (C) and MERS-CoV (D).

(E) Graphs show antiviral activity measured with a SARS-CoV-2 immunostaining signal used for identification of infected A549-ACE2 cells. IC_{50} values were calculated by non-linear regression sigmoidal dose-response analysis using the GraphPad Prism 7 software package.

(F) Graph shows synergistic effect of berzosertib and remdesivir in infected A549-ACE2 cells. Dose-response curves obtained with mixtures of remdesivir and berzosertib, remdesivir alone (thick black line), and berzosertib alone (thick pink line) are shown.

(G) Isobologram of drug combinations is depicted.

(H) Combinatorial data were analyzed for inhibitory, additive, or synergistic effects (upper triangle, dotted line, and lower triangle, respectively) by using the Compusyn software package.

CHK1 (phospho-CHK1-S345) (Hall et al., 2014; Sanjiv et al., 2016). It is known that many viruses hijack that pathway for efficient replication (Boichuk et al., 2010; Lilley et al., 2007), and that pathway is also modulated by DNA tumor viruses (Pancholi et al., 2017). For example, alphaherpesvirus and BK polyomavirus (BKPyV) have been shown to induce the DDR pathway during infection (Lian et al., 2019; Verhalen et al., 2015). It is apparent that many RNA viruses can induce significant DNA damage, even in cases in which viral replication takes place exclusively in the cytoplasm (Ryan et al., 2016; Nguyen et al., 2018). In addition, activation of DDR pathways can contribute positively to replication of viral RNA genomes by switching off cell division, which frees up resources for viral replication. Importantly, studies have shown that SARS-CoV-1 induces DNA replication stress pathway partly by interacting with the p125 subunit of DNA polymerase delta through its nonstructural protein (Zou et al., 2020; Xu et al., 2011). In our research, we observed that SARS-CoV-2 induces phosphorylation of CHK1, a phosphorylation substrate and effector protein of ATR in primary proximal lung epithelial cells (Figure S5E). We observed that Vero E6 cells had a higher basal level of pCHK1; however, treatment with berzosertib reduced phosphorylation of CHK1 and inhibited SARS-CoV-2 infection (Figures 4C and S5E).

Interestingly, another ATR inhibitor AZD6738, did not have an inhibitory effect on SARS-CoV-2 (Figure S5F). Although this fact complicates the picture, it does not necessarily exclude the on-target effect of berzosertib on ATR as a mode of action. The structures of the other ATR inhibitor tested and berzosertib are quite different, and the binding modes and likely binding sites of the inhibitors are different. Moreover, although one may

CellPress
OPEN ACCESS

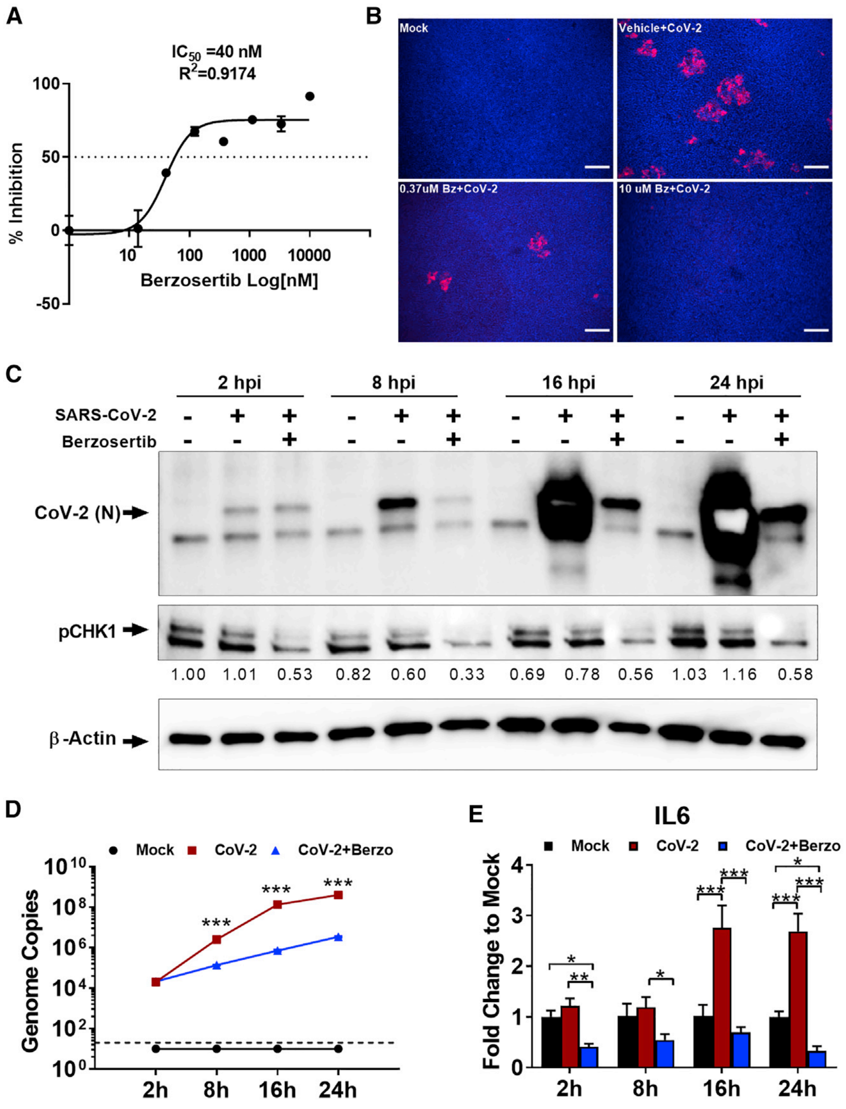

Figure 4. Berzosertib mode of antiviral activity in lung and kidney epithelial cells and effect on SARS-CoV-2-mediated inflammatory response

(A) Graph shows eight-dose-response curve of berzosertib in SARS-CoV-2-infected human primary lung ALI culture.

(B) Immunofluorescent images indicate dose-dependent reduction of SARS-CoV-2 replication in berzosertib-treated ALI culture (spike protein in red).Scale bar, 100 μm.

(C) Western blot analysis shows time course of pCHK1 and virus replication kinetics in Vero E6 cells. Berzosertib treatment reduced CHK1 phosphorylation. In addition, it inhibited SARS-CoV-2 replication as early as 8 h after infection. By 24 h in untreated cells, SARS-CoV-2 signal intensity was oversaturated because of the high-level of viral proteins. Representative data from two independent experiments is shown.

(D) SARS-CoV-2 genome replication kinetics in the presence of berzosertib treatment on Vero E6 cells.

(E) Graph shows that berzosertib treatment reduces the expression of inflammatory IL-6 gene in SARS-CoV-2-infected Vero E6 cells.

Statistical analysis of graphs (D and E) conducted with multiple-comparison two-way analysis of variance (ANOVA). *$p < 0.01$, **$p < 0.001$, ***$p < 0.0001$. Representative data from three independent experiments are presented.

expect an IC_{50} on, e.g., viral RNA replication in the same range as the IC_{50} of the ATR kinase inhibition by berzosertib, this is, by no means, assured. It has to be taken into consideration that the ATR pathway is activated by SARS-CoV-2, which will have its effects on the IC_{50}. Our STRING (search tool for the retrieval of interacting genes/proteins) analysis places the ATR pathway into direct context with, e.g., the mTOR axis (Figure 1), which is known to be vital for SARS CoV-2 pathophysiology. Thus, berzosertib activity is consistent with an on-target mode of action. However, it is possible that the berzosertib anti-SARS-CoV-2 activity might be directly targeted toward viral factors. Differing antiviral activity could also be due to inhibition of additional other cellular targets, because, e.g., berzosertib has an inhibitory effect on DYRK2 and AXL. DYRK2 has been shown previously to negatively regulate type I interferon induction, and a knockdown of DYRK2 has been shown to significantly inhibit vesicular stomatitis virus (VSV) and herpes simplex virus 1 (HSV-1) replication (An et al., 2015); in addition, AXL inhibition can lead to an enhanced antiviral state of cells (Strange et al., 2019). It has been shown that DNA damage can induce a type-I IFN signaling response (Brzostek-Racine et al., 2011). Studies have revealed that inhibition of ATM or ATR can lead to immune stimulation (Sun et al., 2018; Zhang et al., 2019). ATR inhibition for berzosertib has an IC_{50} of 19 nM in HT29 cells (according to the vendor Selleckchem). We observed an IC_{50} of 0.04 μM and 0.48 μM in human primary lung cells and Calu-3 cells, respectively, with no detectable toxicity up to 50 μM in either cell culture. Thus, our data show that, overall, berzosertib has a good therapeutic window.

We further evaluated the characteristics of the SARS CoV-2 inhibition of berzosertib via kinetic experiments. Our data indicate that berzosertib is not an entry inhibitor because SARS-CoV-2 was able to enter, and the number of viral genome copies at 2 hpi in Vero E6 cells was not affected. However, from 8 h onward, both viral genome and protein production were significantly reduced (Figures 4C and 4D). These observations suggest that viral transcription and replication machinery might be inhibited. We are currently examining the mode of action through RNA sequencing (RNA-seq) as well as other methods. Elucidation of the interactions between RNA viruses and the DDR pathway would provide important insights into the modulation of host cell functions by these pathogens.

As the inflammatory host response drives much of the pathology of the SARS CoV-2 infection, it is important to note that berzosertib treatment resulted in reduced viral-mediated induction of the pro-inflammatory response gene IL6 (Figure 4E). Thus, we expect berzosertib to be useful in the treatment of individuals with ongoing infections. As of December 2020, there have been nine oncology clinical studies (at phases 1 and 2) (Konstantinopoulos et al., 2020; Yap et al., 2020; Thomas et al., 2018) based on berzosertib, which have shown that berzosertib is well tolerated in a cancer setting. Although toxicity considered acceptable in that setting, it may not be acceptable in other pathological conditions. Moreover, current NIH COVID-19 treatment guidelines discourage the use of, e.g., JAK inhibitors because they are immunosuppressive. In contrast, berzosertib is an excellent candidate for rapid repurposing toward treating patients with COVID-19 because it does not have, e.g., the immunosuppressive/thrombotic side effects of JAK/STAT inhibitors, which are contraindicated in an infectious disease context (Mehta et al., 2020). In fact, berzosertib is unique in its mode of action because it is intended as a potentiator of the therapeutic effects of genotoxic drugs in oncology, i.e., it is not a standalone therapy. In addition, non-ATR kinase inhibitors can have a much stronger direct effect on cell viability; thus, one can speculate that their adverse side effects in severely affected COVID-19 patients may be much more pronounced than that of berzosertib.

Overall, this present study highlights a potential therapeutic option for SARS-CoV-2 infection and key proteins involved in signaling pathways critical for SARS-CoV-2 replication. The study also provides different avenues for host-directed therapeutic interventions for treatment of patients with COVID-19 with berzosertib as an important addition. Further *in vitro* studies, focusing on a combination of drug candidates blocking key signaling pathways identified in this report, are underway.

STAR★METHODS

Detailed methods are provided in the online version of this paper and include the following:

- KEY RESOURCES TABLE
- RESOURCE AVAILABILITY
  - Lead contact
  - Materials availability
  - Data and code availability
- EXPERIMENTAL MODEL AND SUBJECT DETAILS
  - Cells
  - Virus
  - Drug library and compounds
  - Biosafety and IRB Approval
- METHOD DETAILS
  - SARS-CoV-2 Infection
  - Antiviral Drug Study
  - Viral Titer by TCID50 (Median Tissue Culture Infectious Dose) assay
  - Cytotoxicity Assay
  - Viral replication kinetics
  - Plaque test
  - Cell viability assay
  - High-content screening assay
  - HeLa-ACE2 Uninfected host cell cytotoxicity counter screen
  - Data analysis of SARS-CoV-2/HeLa-ACE2 Experiments
  - Image Analysis/Quantification
  - Immunohistochemistry
  - RNA sample preparation and RT-qPCR
  - Western Blot analysis
- QUANTIFICATION AND STATISTICAL ANALYSIS

SUPPLEMENTAL INFORMATION

Supplemental information can be found online at https://doi.org/10.1016/j.celrep.2021.108940.

ACKNOWLEDGMENTS

This research was funded by many entities as follows: Merck KGaA, Darmstadt, Germany to U.A.K.B., G.G., and R.B.; the California NanoSystems Institute (CNSI) and the Broad Stem Cell Research Center institutional award (OCRC 20-14) to R.D.D.; the UCLA David Geffen School of Medicine (DGSOM) and Broad Stem Cell Research Center institutional award (OCRC 20-15) and the National Eye Institute of the NIH award (1R01EY032149) to V.A.; the California Institute for Regenerative Medicine Discovery Awards (DISC2COVID19-11764 to B.G and TRAN1COVID19-11975 to V.A.); an institutional training grant (T32 HL116273) to A.S.; and an American Heart Association COVID-19 Rapid Response Grant (AHA 814630) to A.S and C.N.S. The research was supported by the UCLA Molecular Screening Shared Resource which is supported by the California NanoSystems Institute and the Jonsson Comprehensive Cancer Center, award number P30CA016042, by the National Cancer Institute of the NIH. Work at Calibr was supported by grants from the Bill & Melinda Gates Foundation (OPP1208899 and INV-018812). The following reagents were obtained through BEI Resources, NIAID, NIH: monoclonal anti-SARS-CoV S protein (similar to 240C), NR-616, and polyclonal anti-SARS coronavirus (antiserum, guinea pig), NR-10361. The following reagent was deposited by the Centers for Disease Control and Prevention and obtained through BEI Resources, NIAID, NIH: SARS-related coronavirus 2, isolate USA-WA1/2020, NR-52281. We thank David Austin for cell quantification at the Molecular Screening Shared Resource (MSSR) at UCLA. We thank Kouki Morizono for providing the 293T-ACE2 cell line. We are grateful to Barbara Dillon, UCLA High-Containment Program Director for BSL3 work. We are grateful to Dirk Finsinger, Sven Poetzsch, Markus B. Klein, Friedrich Rippmann, Anna Coenen-Stass, Thomas Fuchss, Lukas Friedrich, Frank T. Zenke, Arno Hartmann, Stephanie Josupeit, Peter Pokinskyj, Robert Garces, Jörg Zissel, Jan-Carsten Pieck, Frederique Santerre, Joern-Peter Halle, Belen Garijo, and Stefan Oschmann for supporting the project at Merck KGaA. We are grateful to the Calibr Compound Management and High Throughput Screening Groups, including Emily Chen, Tu-Trinh Nguyen, Alonzo Davila, Hannah Hoang, and Mitchell V. Hull for logistics, compound handling, and assay plate preparation. We thank Marc Roseboro, Digital Communications Manager (CNSI), for assisting in developing the graphical abstract.

AUTHOR CONTRIBUTIONS

G. Garcia conceived and designed the study, collected and/or assembled data, analyzed and interpreted data, and wrote the manuscript. A.S., C.S., and A.P. conducted experiments and analyzed and interpreted data. A.R., D.B.K., M.S.P., B.G., D.U., P.K.P., P.S., N.B., T.F.R., A.K.C., and C.N.S. provided experimental design, analyzed and interpreted data, and wrote the manuscript. G. Gabriel provided conception and design. S.B., H.K., M.A.B., M.G.K., L.R., K.C.W., B.H., and C.Y. conducted experiments and analyzed and interpreted data. R.B. provided data analysis and interpretation and final approval of the manuscript. U.A.K.B., R.D.D., and V.A. conceived and

CellPress
OPEN ACCESS

designed the study, analyzed and interpreted data, wrote the manuscript, and provided final approval of the manuscript.

DECLARATION OF INTERESTS

U.A.K.B. is an employee of Merck KGaA, Darmstadt, Germany. Berzosertib compound is licensed by Merck KGaA, Darmstadt, Germany. The other authors declare no competing financial interests.

Received: August 22, 2020
Revised: January 26, 2021
Accepted: March 12, 2021
Published: March 18, 2021

REFERENCES

An, T., Li, S., Pan, W., Tien, P., Zhong, B., Shu, H.B., and Wu, S. (2015). DYRK2 negatively regulates type I interferon induction by promoting TBK1 degradation via Ser527 phosphorylation. PLoS Pathog. *11*, e1005179.

Belhadjer, Z., Méot, M., Bajolle, F., Khraiche, D., Legendre, A., Abakka, S., Auriau, J., Grimaud, M., Oualha, M., Beghetti, M., et al. (2020). Acute heart failure in multisystem inflammatory syndrome in children (MIS-C) in the context of global SARS-CoV-2 pandemic. Circulation *142*, 429–436.

Boichuk, S., Hu, L., Hein, J., and Gjoerup, O.V. (2010). Multiple DNA damage signaling and repair pathways deregulated by simian virus 40 large T antigen. J. Virol. *84*, 8007–8020.

Bose, S.K., Shrivastava, S., Meyer, K., Ray, R.B., and Ray, R. (2012). Hepatitis C virus activates the mTOR/S6K1 signaling pathway in inhibiting IRS-1 function for insulin resistance. J. Virol. *86*, 6315–6322.

Brzostek-Racine, S., Gordon, C., Van Scoy, S., and Reich, N.C. (2011). The DNA damage response induces IFN. J. Immunol. *187*, 5336–5345.

Campbell, G.R., Bruckman, R.S., Herns, S.D., Joshi, S., Durden, D.L., and Spector, S.A. (2018). Induction of autophagy by PI3K/MTOR and PI3K/MTOR/BRD4 inhibitors suppresses HIV-1 replication. J. Biol. Chem. *293*, 5808–5820.

Chou, T.C. (2006). Theoretical basis, experimental design, and computerized simulation of synergism and antagonism in drug combination studies. Pharmacol. Rev. *58*, 621–681.

Coleman, D.T., Gray, A.L., Stephens, C.A., Scott, M.L., and Cardelli, J.A. (2016). Repurposed drug screen identifies cardiac glycosides as inhibitors of TGF-β-induced cancer-associated fibroblast differentiation. Oncotarget *7*, 32200–32209.

Craver, R., Huber, S., Sandomirsky, M., McKenna, D., Schieffelin, J., and Finger, L. (2020). Fatal eosinophilic myocarditis in a healthy 17-year-old male with severe acute respiratory syndrome coronavirus 2 (SARS-CoV-2c). Fetal Pediatr. Pathol. *39*, 263–268.

Dolhnikoff, M., Ferreira ferranti, J., de Almeida Monteiro, R.A., Duarte-Neto, A.N., Soares Gomes-Gouvêa, M., Viu Degaspare, N., Figueiredo Delgado, A., Montanari Fiorita, C., Nunes Leal, G., Rodrigues, R.M., et al. (2020). SARS-CoV-2 in cardiac tissue of a child with COVID-19-related multisystem inflammatory syndrome. Lancet Child Adolesc. Health *4*, 790–794.

Dong, E., Du, H., and Gardner, L. (2020). An interactive web-based dashboard to track COVID-19 in real time. Lancet Infect. Dis. *20*, 533–534.

Dyall, J., Coleman, C.M., Hart, B.J., Venkataraman, T., Holbrook, M.R., Kindrachuk, J., Johnson, R.F., Olinger, G.G., Jr., Jahrling, P.B., Laidlaw, M., et al. (2014). Repurposing of clinically developed drugs for treatment of Middle East respiratory syndrome coronavirus infection. Antimicrob. Agents Chemother. *58*, 4885–4893.

Escher, F., Pietsch, H., Aleshcheva, G., Bock, T., Baumeier, C., Elsaesser, A., Wenzel, P., Hamm, C., Westenfeld, R., Schultheiss, M., et al. (2020). Detection of viral SARS-CoV-2 genomes and histopathological changes in endomyocardial biopsies. ESC Heart Fail. *7*, 2440–2447.

Fanelli, V., Fiorentino, M., Cantaluppi, V., Gesualdo, L., Stallone, G., Ronco, C., and Castellano, G. (2020). Acute kidney injury in SARS-CoV-2 infected patients. Crit. Care *24*, 155.

Fried, J.A., Ramasubbu, K., Bhatt, R., Topkara, V.K., Clerkin, K.J., Horn, E., Rabbani, L., Brodie, D., Jain, S.S., Kirtane, A.J., et al. (2020). The Variety of Cardiovascular Presentations of COVID-19. Circulation *141*, 1930–1936.

García, M., Cooper, A., Shi, W., Bornmann, W., Carrion, R., Kalman, D., and Nabel, G.J. (2012). Productive replication of Ebola virus is regulated by the c-Abl1 tyrosine kinase. Sci. Transl. Med. *4*, 123ra24.

Gauger, P.C., and Vincent, A.L. (2014). Serum virus neutralization assay for detection and quantitation of serum-neutralizing antibodies to influenza A virus in swine. Methods Mol. Biol. *1161*, 313–324.

Gnecchi, M., Moretti, F., Bassi, E.M., Leonardi, S., Totaro, R., Perotti, L., Zuccaro, V., Perlini, S., Preda, L., Baldanti, F., et al. (2020). Myocarditis in a 16-year-old boy positive for SARS-CoV-2. Lancet *395*, e116.

Grimaud, M., Starck, J., Levy, M., Marais, C., Chareyre, J., Khraiche, D., Leruez-Ville, M., Quartier, P., Léger, P.L., Geslain, G., et al. (2020). Acute myocarditis and multisystem inflammatory emerging disease following SARS-CoV-2 infection in critically ill children. Ann. Intensive Care *10*, 69.

Gross, S., Rahal, R., Stransky, N., Lengauer, C., and Hoeflich, K.P. (2015). Targeting cancer with kinase inhibitors. J. Clin. Invest. *125*, 1780–1789.

Hall, A.B., Newsome, D., Wang, Y., Boucher, D.M., Eustace, B., Gu, Y., Hare, B., Johnson, M.A., Milton, S., Murphy, C.E., et al. (2014). Potentiation of tumor responses to DNA damaging therapy by the selective ATR inhibitor VX-970. Oncotarget *5*, 5674–5685.

Hou, Y.J., Okuda, K., Edwards, C.E., Martinez, D.R., Asakura, T., Dinnon, K.H., Kato, T., Lee, R.E., Yount, B.L., Mascenik, T.M., et al. (2020). SARS-CoV-2 reverse genetics reveals a variable infection gradient in the respiratory tract. Cell *182*, 429–446.e14.

Jeon, S., Ko, M., Lee, J., Choi, I., Byun, S.Y., Park, S., Shum, D., and Kim, S. (2020). Identification of antiviral drug candidates against SARS-CoV-2 from FDA-approved drugs. Antimicrob. Agents Chemother. *64*, e00819–e00820.

Jiang, W.M., Zhang, X.Y., Zhang, Y.Z., Liu, L., and Lu, H.Z. (2014). A high throughput RNAi screen reveals determinants of HIV-1 activity in host kinases. Int. J. Clin. Exp. Pathol. *7*, 2229–2237.

Johansen, L.M., Brannan, J.M., Delos, S.E., Shoemaker, C.J., Stossel, A., Lear, C., Hoffstrom, B.G., Dewald, L.E., Schornberg, K.L., Scully, C., et al. (2013). FDA-approved selective estrogen receptor modulators inhibit Ebola virus infection. Sci. Transl. Med. *5*, 190ra79.

Kai, H., and Kai, M. (2020). Interactions of coronaviruses with ACE2, angiotensin II, and RAS inhibitors-lessons from available evidence and insights into COVID-19. Hypertens. Res. *43*, 648–654.

Keating, J.A., and Striker, R. (2012). Phosphorylation events during viral infections provide potential therapeutic targets. Rev. Med. Virol. *22*, 166–181.

Kong, K., Kumar, M., Taruishi, M., and Javier, R.T. (2014). The human adenovirus E4-ORF1 protein subverts discs large 1 to mediate membrane recruitment and dysregulation of phosphatidylinositol 3-kinase. PLoS Pathog. *10*, e1004102.

Konstantinopoulos, P.A., Cheng, S.C., Wahner Hendrickson, A.E., Penson, R.T., Schumer, S.T., Doyle, L.A., Lee, E.K., Kohn, E.C., Duska, L.R., Crispens, M.A., et al. (2020). Berzosertib plus gemcitabine versus gemcitabine alone in platinum-resistant high-grade serous ovarian cancer: a multicentre, open-label, randomised, phase 2 trial. Lancet Oncol. *21*, 957–968.

Kuhn, M., von Mering, C., Campillos, M., Jensen, L.J., and Bork, P. (2008). STITCH: interaction networks of chemicals and proteins. Nucleic Acids Res. *36*, D684–D688.

Kuss-Duerkop, S.K., Wang, J., Mena, I., White, K., Metreveli, G., Sakthivel, R., Mata, M.A., Muñoz-Moreno, R., Chen, X., Krammer, F., et al. (2017). Influenza virus differentially activates mTORC1 and mTORC2 signaling to maximize late stage replication. PLoS Pathog. *13*, e1006635.

Lan, F., Lee, A.S., Liang, P., Sanchez-Freire, V., Nguyen, P.K., Wang, L., Han, L., Yen, M., Wang, Y., Sun, N., et al. (2013). Abnormal calcium handling

properties underlie familial hypertrophic cardiomyopathy pathology in patient-specific induced pluripotent stem cells. Cell Stem Cell *12*, 101–113.

Le Sage, V., Cinti, A., Amorim, R., and Mouland, A.J. (2016). Adapting the stress response: viral subversion of the mTOR signaling pathway. Viruses *8*, 152.

Li, Q., Brass, A.L., Ng, A., Hu, Z., Xavier, R.J., Liang, T.J., and Elledge, S.J. (2009). A genome-wide genetic screen for host factors required for hepatitis C virus propagation. Proc. Natl. Acad. Sci. USA *106*, 16410–16415.

Li, M.-Y., Li, L., Zhang, Y., and Wang, X.-S. (2020). Expression of the SARS-CoV-2 cell receptor gene ACE2 in a wide variety of human tissues. Infect. Dis. Poverty *9*, 45.

Lian, X., Bao, C., Li, X., Zhang, X., Chen, H., Jung, Y.S., and Qian, Y. (2019). Marek's disease virus disables the ATR-Chk1 pathway by activating STAT3. J. Virol. *93*, e02290-18.

Lilley, C.E., Schwartz, R.A., and Weitzman, M.D. (2007). Using or abusing: viruses and the cellular DNA damage response. Trends Microbiol. *15*, 119–126.

Liu, J., Cao, R., Xu, M., Wang, X., Zhang, H., Hu, H., Li, Y., Hu, Z., Zhong, W., and Wang, M. (2020). Hydroxychloroquine, a less toxic derivative of chloroquine, is effective in inhibiting SARS-CoV-2 infection in vitro. Cell Discov. *6*, 16.

Lu, Y., Li, X., Geng, D., Mei, N., Wu, P.-Y., Huang, C.-C., Jia, T., Zhao, Y., Wang, D., Xiao, A., et al. (2020). Cerebral micro-structural changes in COVID-19 patients—an MRI-based 3-month follow-up study. EClinicalMedicine *25*, 100484.

Madrid, P.B., Chopra, S., Manger, I.D., Gilfillan, L., Keepers, T.R., Shurtleff, A.C., Green, C.E., Iyer, L.V., Dilks, H.H., Davey, R.A., et al. (2013). A systematic screen of FDA-approved drugs for inhibitors of biological threat agents. PLoS ONE *8*, e60579.

Mehta, P., Ciurtin, C., Scully, M., Levi, M., and Chambers, R.C. (2020). JAK inhibitors in COVID-19: the need for vigilance regarding increased inherent thrombotic risk. Eur. Respir. J. *56*, 2001919.

Mei, L., Zhang, J., He, K., and Zhang, J. (2019). Ataxia telangiectasia and Rad3-related inhibitors and cancer therapy: where we stand. J. Hematol. Oncol. *12*, 43.

Moody, C.A., Scott, R.S., Amirghahari, N., Nathan, C.O., Young, L.S., Dawson, C.W., and Sixbey, J.W. (2005). Modulation of the cell growth regulator mTOR by Epstein-Barr virus-encoded LMP2A. J. Virol. *79*, 5499–5506.

Nguyen, T.T.T., Park, E.-M., Lim, Y.-S., and Hwang, S.B. (2018). Nonstructural protein 5A impairs DNA damage repair: implications for hepatitis C virus-mediated hepatocarcinogenesis. J. Virol. *92*, e00178-18.

Ott, P.A., and Adams, S. (2011). Small-molecule protein kinase inhibitors and their effects on the immune system: implications for cancer treatment. Immunotherapy *3*, 213–227.

Pacciarini, F., Ghezzi, S., Canducci, F., Sims, A., Sampaolo, M., Ferioli, E., Clementi, M., Poli, G., Conaldi, P.G., Baric, R., and Vicenzi, E. (2008). Persistent replication of severe acute respiratory syndrome coronavirus in human tubular kidney cells selects for adaptive mutations in the membrane protein. J. Virol. *82*, 5137–5144.

Pancholi, N.J., Price, A.M., and Weitzman, M.D. (2017). Take your PIKK: tumour viruses and DNA damage response pathways. Philos. Trans. R. Soc. Lond. B Biol. Sci. *372*, 20160269.

Paterson, R.W., Brown, R.L., Benjamin, L., Nortley, R., Wiethoff, S., Bharucha, T., Jayaseelan, D.L., Kumar, G., Raftopoulos, R.E., Zambreanu, L., et al. (2020). The emerging spectrum of COVID-19 neurology: clinical, radiological and laboratory findings. Brain *143*, 3104–3120.

Puelles, V.G., Lütgehetmann, M., Lindenmeyer, M.T., Sperhake, J.P., Wong, M.N., Allweiss, L., Chilla, S., Heinemann, A., Wanner, N., Liu, S., et al. (2020). α N. Engl. J. Med. 383, 590–592.

Purkayastha, A., Sen, C., Garcia, G., Jr., Langerman, J., Shia, D.W., Meneses, L.K., Vijayaraj, P., Durra, A., Koloff, C.R., Freund, D.R., et al. (2020). Direct exposure to SARS-CoV-2 and cigarette smoke increases infection severity and alters the stem cell-derived airway repair response. Cell Stem Cell *27*, 869–875.e4.

Ramaiah, A., and Arumugaswami, V. (2020). Insights into cross-species evolution of novel human coronavirus 2019-nCoV and defining immune determinants for vaccine development. bioRxiv, 2020.01.29.925867.

Riva, L., Yuan, S., Yin, X., Martin-Sancho, L., Matsunaga, N., Burgstaller-Muehlbacher, S., Pache, L., De Jesus, P.P., Hull, M.V., Chang, M., et al. (2020). A large-scale drug repositioning survey for SARS-CoV-2 antivirals. bioRxiv, 2020.04.16.044016.

Ryan, E.L., Hollingworth, R., and Grand, R.J. (2016). Activation of the DNA damage response by RNA viruses. Biomolecules *6*, 2.

Sanjiv, K., Hagenkort, A., Calderón-Montaño, J.M., Koolmeister, T., Reaper, P.M., Mortusewicz, O., Jacques, S.A., Kuiper, R.V., Schultz, N., Scobie, M., et al. (2016). Cancer-specific synthetic lethality between ATR and CHK1 kinase activities. Cell Rep. *14*, 298–309.

Sanna, G., Serrau, G., Bassareo, P.P., Neroni, P., Fanos, V., and Marcialis, M.A. (2020). Children's heart and COVID-19: Up-to-date evidence in the form of a systematic review. Eur. J. Pediatr. *179*, 1079–1087.

Schor, S., and Einav, S. (2018). Repurposing of kinase inhibitors as broad-spectrum antiviral drugs. DNA Cell Biol. *37*, 63–69.

Sharma, A., Li, G., Rajarajan, K., Hamaguchi, R., Burridge, P.W., and Wu, S.M. (2015). Derivation of highly purified cardiomyocytes from human induced pluripotent stem cells using small molecule-modulated differentiation and subsequent glucose starvation. J. Vis. Exp. (*97*), 52628.

Sharma, A., Garcia, G., Arumugaswami, V., and Svendsen, C.N. (2020). Human iPSC-derived cardiomyocytes are susceptible to SARS-CoV-2 infection. Cell Rep. Med. *1*, 1000052.

Shi, S., Qin, M., Shen, B., Cai, Y., Liu, T., Yang, F., Gong, W., Liu, X., Liang, J., Zhao, Q., et al. (2020). Association of cardiac injury with mortality in hospitalized patients with COVID-19 in Wuhan, China. JAMA Cardiol. *5*, 802–810.

Sodhi, A., Chaisuparat, R., Hu, J., Ramsdell, A.K., Manning, B.D., Sausville, E.A., Sawai, E.T., Molinolo, A., Gutkind, J.S., and Montaner, S. (2006). The TSC2/mTOR pathway drives endothelial cell transformation induced by the Kaposi's sarcoma-associated herpesvirus G protein-coupled receptor. Cancer Cell *10*, 133–143.

Strange, D.P., Jiyarom, B., Pourhabibi Zarandi, N., Xie, X., Baker, C., Sadri-Ardekani, H., Shi, P.Y., and Verma, S. (2019). Axl promotes Zika virus entry and modulates the antiviral state of human Sertoli cells. MBio *10*, e01372-19.

Sun, N., Yazawa, M., Liu, J., Han, L., Sanchez-Freire, V., Abilez, O.J., Navarrete, E.G., Hu, S., Wang, L., Lee, A., et al. (2012). Patient-specific induced pluripotent stem cells as a model for familial dilated cardiomyopathy. Sci. Transl. Med. *4*, 130ra47.

Sun, L.L., Yang, R.Y., Li, C.W., Chen, M.K., Shao, B., Hsu, J.M., Chan, L.C., Yang, Y., Hsu, J.L., Lai, Y.J., and Hung, M.C. (2018). Inhibition of ATR down-regulates PD-L1 and sensitizes tumor cells to T cell-mediated killing. Am. J. Cancer Res. *8*, 1307–1316.

Supekova, L., Supek, F., Lee, J., Chen, S., Gray, N., Pezacki, J.P., Schlapbach, A., and Schultz, P.G. (2008). Identification of human kinases involved in hepatitis C virus replication by small interference RNA library screening. J. Biol. Chem. *283*, 29–36.

Tavazzi, G., Pellegrini, C., Maurelli, M., Belliato, M., Sciutti, F., Bottazzi, A., Sepe, P.A., Resasco, T., Camporotondo, R., Bruno, R., et al. (2020). Myocardial localization of coronavirus in COVID-19 cardiogenic shock. Eur. J. Heart Fail. *22*, 911–915.

Thomas, A., Redon, C.E., Sciuto, L., Padiernos, E., Ji, J., Lee, M.J., Yuno, A., Lee, S., Zhang, Y., Tran, L., et al. (2018). Phase I Study of ATR Inhibitor M6620 in Combination With Topotecan in Patients With Advanced Solid Tumors. J. Clin. Oncol. *36*, 1594–1602.

Tufts Center for the Study of Drug Development (2014). Cost to develop and win 279 marketing approval for a new drug is $2.6 billion.

Varga, Z., Flammer, A.J., Steiger, P., Haberecker, M., Andermatt, R., Zinkernagel, A.S., Mehra, M.R., Schuepbach, R.A., Ruschitzka, F., and Moch, H. (2020). Endothelial cell infection and endotheliitis in COVID-19. Lancet *395*, 1417–1418.

CellPress
OPEN ACCESS

Verhalen, B., Justice, J.L., Imperiale, M.J., and Jiang, M. (2015). Viral DNA replication-dependent DNA damage response activation during BK polyomavirus infection. J. Virol. *89*, 5032–5039.

Weichhart, T. (2012). Mammalian target of rapamycin: a signaling kinase for every aspect of cellular life. Methods Mol. Biol. *821*, 1–14.

Weisberg, E., Parent, A., Yang, P.L., Sattler, M., Liu, Q., Liu, Q., Wang, J., Meng, C., Buhrlage, S.J., Gray, N., and Griffin, J.D. (2020). Repurposing of kinase inhibitors for treatment of COVID-19. Pharm. Res. *37*, 167.

Weston, S., Coleman, C.M., Haupt, R., Logue, J., Matthews, K., Li, Y., Reyes, H.M., Weiss, S.R., and Frieman, M.B. (2020). Broad anti-coronavirus activity of Food and Drug Administration-approved drugs against SARS-CoV-2 *in vitro* and SARS-CoV *in vivo*. J. Virol. *94*, e01218–e01220.

WORLDOMETER (2020). What are the sources for the coronavirus COVID-19 numbers?.

Xu, L.H., Huang, M., Fang, S.G., and Liu, D.X. (2011). Coronavirus infection induces DNA replication stress partly through interaction of its nonstructural protein 13 with the p125 subunit of DNA polymerase δ. J. Biol. Chem. *286*, 39546–39559.

Xu, H., Zhong, L., Deng, J., Peng, J., Dan, H., Zeng, X., Li, T., and Chen, Q. (2020a). High expression of ACE2 receptor of 2019-nCoV on the epithelial cells of oral mucosa. Int. J. Oral Sci. *12*, 8.

Xu, Z., Shi, L., Wang, Y., Zhang, J., Huang, L., Zhang, C., Liu, S., Zhao, P., Liu, H., Zhu, L., et al. (2020b). Pathological findings of COVID-19 associated with acute respiratory distress syndrome. Lancet Respir. Med. *8*, 420–422.

Yancy, C.W., and Fonarow, G.C. (2020). Coronavirus disease 2019 (COVID-19) and the heart—is heart failure the next chapter? JAMA Cardiol. *5*, 1216–1217.

Yap, T.A., O'Carrigan, B., Penney, M.S., Lim, J.S., Brown, J.S., de Miguel Luken, M.J., Tunariu, N., Perez-Lopez, R., Rodrigues, D.N., Riisnaes, R., et al. (2020). Phase I trial of first-in-class ATR inhibitor M6620 (VX-970) as monotherapy or in combination with carboplatin in patients with advanced solid tumors. J. Clin. Oncol. *38*, 3195–3204.

Zhang, Q., Green, M.D., Lang, X., Lazarus, J., Parsels, J.D., Wei, S., Parsels, L.A., Shi, J., Ramnath, N., Wahl, D.R., et al. (2019). Inhibition of ATM increases interferon signaling and sensitizes pancreatic cancer to immune checkpoint blockade therapy. Cancer Res. *79*, 3940–3951.

Zou, X., Chen, K., Zou, J., Han, P., Hao, J., and Han, Z. (2020). Single-cell RNA-seq data analysis on the receptor ACE2 expression reveals the potential risk of different human organs vulnerable to 2019-nCoV infection. Front. Med. *14*, 185–192.

STAR★METHODS

KEY RESOURCES TABLE

REAGENT or RESOURCE	SOURCE	IDENTIFIER
**Antibodies**		
Monoclonal anti-SARS-CoV S protein (Similar to 240C) antibody	BEI Resources Repository	Cat#NR-616
Polyclonal anti-SARS coronavirus (antiserum)	BEI Resources Repository	Cat#NR-10361
Monoclonal anti-dsRNA antibody (J2 clone)	Absolute Antibody	Cat#Ab01299-2.0
Cleaved caspase-3 rabbit monoclonal antibody, clone D175	Cell Signaling	Cat#9661S; RRID: AB_2341188
Goat anti-Mouse IgG (H+L) Cross-Adsorbed Secondary Antibody, Alexa Fluor 555	Thermo Fisher Scientific	Cat#A21422; RRID: AB_2535844
IgG (H+L) Cross-Adsorbed Goat anti-Rabbit, Alexa Fluor 488, Invitrogen	Thermo Fisher Scientific	Cat#A11008; RRID: AB_143165
IgG (H+L) Cross-Adsorbed Goat anti-Human, Alexa Fluor 488, Invitrogen	Thermo Fisher Scientific	Cat#A11013; RRID: AB_2534080
Phospho-Chk1 (Ser345) (133D3) Rabbit mAb	Cell Signaling	Cat#2348; RRID: AB_331212
Monoclonal Anti-Beta-Actin, Clone AC-74 produced in mouse	MilliporeSigma	Cat#A2228; RRID: AB_476697
Phospho-Stat1 (Tyr701) (58D6) Rabbit mAb	Cell Signaling	Cat#9167S; RRID: AB_561284
Stat1 (D1K9Y) Rabbit mAb	Cell Signaling	Cat#14994; RRID: AB_2737027
**Bacterial and Virus Strains**		
SARS-Related Coronavirus 2 (SARS-CoV-2), Isolate USA-WA1/2020	BEI Resources Repository	Cat#NR-52281
SARS-Associated Coronavirus (SARS-CoV), Strain Frankfurt 1	Dr. B. L. Haagmans (Erasmus Medical Center, Rotterdam, the Netherlands)	N/A
MERS-CoV, Strain EMC-2012	Dr. Christian Drosten (Charité Universitätsmedizin Berlin, Berlin, Germany)	N/A
SARS-Related Coronavirus 2 (SARS-CoV-2/Germany/ HPI06-n/2020)	University Medical Campus Hamburg-Eppendorf, Hamburg, Germany	N/A
**Chemicals, peptides, and recombinant proteins**		
Regular Fetal Bovine Serum	Corning	Cat#35010CV
Eagle's Minimum Essential Medium (MEM)	Corning	Cat#10009CV
Penicillin-Streptomycin (10,000 U/mL)	GIBCO	Cat#15140122
L-Glutamine (200 mM)	GIBCO	Cat#25030081
Puromycin Dihydrochloride	GIBCO	Cat#A1113803
PneumaCult-ALI Medium	STEMCELL Technologies	Cat#05021
Berzosertib	MERCK KGaA	N/A
Selected Kinase Inhibitor Library	Selleck Chemicals	N/A
Dimethyl sulfoxide	MilliporeSigma	Cat#D2650
RPMI 1640	Thermo Fisher Scientific	Cat#11875093
B27 supplement with insulin	Thermo Fisher Scientific	Cat#17504044
Methanol (Histological)	Thermo Fisher Scientific	Cat#A433P4
16% Paraformaldehyde (formaldehyde) aqueous solution	Electron Microscopy Sciences	Cat#15710
Dulbecco's Phosphate-Buffered Salt Solution 1X	Corning	Cat#21030CV
Perm/Wash Buffer	BD Biosciences	Cat#554723
DAPI (4',6-Diamidino-2-Phenylindole, Dihydrochloride)	Thermo Fisher Scientific	Cat#D1306
SuperBlock (PBS) Blocking Buffer	Thermo Fisher Scientific	Cat#37515

(*Continued on next page*)

CellPress
OPEN ACCESS

Continued

REAGENT or RESOURCE	SOURCE	IDENTIFIER
Bovine Serum Albumin	MilliporeSigma	Cat#A9418
Normal Donkey Serum	Jackson ImmunoResearch	Cat#017-000-121
Normal Goat Serum	Cell Signaling	Cat#5425S
Triton X-100	MilliporeSigma	Cat#T9284
Remdesivir (GS-5734)	Selleck Chemicals	Cat#S8932
Hydroxychloroquine Sulfate	Selleck Chemicals	Cat#S4430
Dactolisib (BEZ235)	Selleck Chemicals	Cat#S1009
Nilotinib (AMN-107)	Selleck Chemicals	Cat#S1033
NVP-BHG712	Selleck Chemicals	Cat#S2202
VPS34-IN1	Selleck Chemicals	Cat#S7980
YM201636	Selleck Chemicals	Cat#S1219
Vistusertib (AZD2014)	Selleck Chemicals	Cat#S2783
AZD8055	Selleck Chemicals	Cat#S1555
URMC099	Selleck Chemicals	Cat#S7343
TORIN2	Selleck Chemicals	Cat#S2817
Critical commercial assays		
MTT Cell Proliferation Assay	ATCC	Cat#30-1010K
CellTiter 96® Non-Radioactive Cell Proliferation Assay (MTT)	Promega	Cat#G4000
Experimental models: Cell lines		
VERO C1008 [Vero 76, clone E6, Vero E6]	ATCC	Cat#CRL-158
Calu-3	ATCC	Cat#HTB-55
HEK293-ACE2	This Paper	N/A
HeLa-ACE2	This Paper	N/A
A549-ACE2	This Paper	N/A
Human induced pluripotent stem cell-derived cardiomyocytes (hiPSC-CMs)	Cedars-Sinai Medical Center (Sharma et al., 2020)	N/A
Normal human bronchial epithelial cells	Lonza	N/A
Oligonucleotides		
Primers for Human GAPDH (Forward:CCACCTTTGACGCTGGG; Reverse:CATACCAGGAAATGAGCTTGACA)	This Paper	N/A
Primers for 2019-nCoV_N1 (Forward:GACCCCAAAATCAGCGAAAT; Reverse:TCTGGTTACTGCCAGTTGAATCTG)	This Paper	N/A
Primers for Human IL6 (Forward:GGAGACTTGCCTGGTGAAA; Reverse:CTGGCTTGTTCCTCACTACTC)	This Paper	N/A
Software and algorithms		
GraphPad Prism 8	GraphPad	N/A
Multi-Point Tool (Cell Counter)	ImageJ	N/A

RESOURCE AVAILABILITY

Lead contact

Further information and requests for resources and reagents should be directed to and will be fulfilled by the Lead Contact, Vaithilingaraja Arumugaswami (varumugaswami@mednet.ucla.edu)

Materials availability

This study did not generate new unique reagents.

Data and code availability

Original data have been deposited to Mendeley Data: [https://doi.org/10.17632/vnwdznrsjb.1].

EXPERIMENTAL MODEL AND SUBJECT DETAILS

Cells

Vero E6 cells were obtained from ATCC [VERO C1008 (ATCC® CRL-1586)] or DSMZ (Braunschweig, Germany). Cells were cultured in EMEM growth media containing 10% fetal bovine serum (FBS) and penicillin (100 units/ml). Human lung adenocarcinoma epithelial cell line (Calu-3) was purchased from ATCC (ATCCHTB-55) and cultured in Dulbecco's Modified Eagles Medium (DMEM), supplemented with 20 % fetal bovine serum (FBS), 1 % L-glutamine (L-glu) and 1 % penicillin/streptomycin (P/S). ACE2 entry receptor overexpressing human embryonic kidney 293 cells (HEK293-ACE2), human cervical epithelial line HeLa (HeLa-ACE2), and human lung epithelial line A549 (A549-ACE2) were established and cultured in the media described above with the presence of puromycin (1 μg/ml). HeLA and A549 cell lines were obtained from ATCC. Cells were incubated at 37°C with 5% CO_2. The hiPSC-CMs were generated from hiPSCs by directed differentiation approach modulating Wnt signaling using a small-molecule (Sharma et al., 2015) and as previously described (Sharma et al., 2015), cardiomyocytes were metabolically selected by using glucose deprivation. After selection, hiPSC-CMs were replated for viral infection. Air-liquid interface (ALI) cultures derived from primary human proximal airway basal stem cells (ABSCs) were used as described previously (Purkayastha et al., 2020). 24-well 6.5mm trans-wells with 0.4mm pore polyester membrane inserts were used for culturing ALI cells. 500 μl ALI media (PneumaCult-ALI Medium, STEMCELL Technologies) was used in the basal chamber for ALI cultures and cells were cultured at 37°C with 5% CO_2.

Virus

SARS-Related Coronavirus 2 (SARS-CoV-2), Isolate USA-WA1/2020, was obtained from BEI Resources of National Institute of Allergy and Infectious Diseases (NIAID). SARS-CoV (Frankfurt 1, FFM) was a kind gift from Dr. B. L. Haagmans (Erasmus Medical Center, Rotterdam, the Netherlands), and MERS-CoV (EMC-2012) was a kind gift from Prof. Dr. Christian Drosten (Charité Universitätsmedizin Berlin, Berlin, Germany). SARS-CoV-2 (SARS-CoV-2/Germany/HPI06-n/2020) was isolated from a nasal swab of a SARS-CoV-2 infected patient who was treated in an ICU at the University Medical Campus Hamburg-Eppendorf, Hamburg, Germany. All the studies involving live virus was conducted in UCLA BSL3 high-containment facility. SARS-CoV-2 was passaged once in Vero E6 cells and viral stocks were aliquoted and stored at −80°C. Virus titer was measured in Vero E6 cells by established plaque assay or TCID50 assay.

Drug library and compounds

The compounds tested for their potential to inhibit SARS-CoV-2 replication and replication of other related CoVs were obtained from MERCK KGaA and Selleck Chemicals. A selected kinase inhibitor library was procured from Selleck Chemicals since this library contains inhibitors for many key kinases. All compounds were provided in DMSO at a final concentration of 10 mM and stored in dark at room temperature or −20°C. Repeated freeze-thaw circles were avoided whenever possible.

Biosafety and IRB Approval

Appropriate institutional review boards (IRB) approvals were obtained at UCLA and Cedars-Sinai Medical Center. All hiPSC lines used in this study have been approved by the UCLA and Cedars-Sinai Medical Center human pluripotent stem cell research oversight committees.

METHOD DETAILS

SARS-CoV-2 Infection

Vero E6 cells were seeded at 5×10^3 cells per well in 0.2 mL volumes using a 96-well plate and hiPSC-CMs were replated at 1×10^5 cells per well. The following day, viral inoculum (MOI of 0.01 and 0.1; 100 μl/well) was prepared using serum free media. The spent media from each well was removed and 100 μl of prepared inoculum was added onto cells. 100 μl of inoculum prepared in PneumaCult media was added to the apical chamber of ALI culture insert. For mock infection, serum free media (100 μl/well) alone was added. The inoculated plates were incubated for 1 hr at 37°C with 5% CO_2. The inoculum was spread by gently tilting the plate sideways at every 15 minutes. At the end of incubation, the inoculum was removed for ALI culture and replaced for Vero E6 cells with serum supplemented media (200 μl per well) and for hiPSC-CM, cell culture medium was replaced with RPMI 1640 + B27 supplement with insulin. At selected time points live cell images were obtained by bright field microscope. At 48 hours post infection (hpi) the cells were fixed with methanol or 4% PFA. Viral infection was examined by immunocytochemistry (ICC) analysis using SARS-CoV Spike (S) antibodies [BEI Resources: NR-10361 polyclonal anti-SARS coronavirus (antiserum, Guinea Pig), and NR-616 monoclonal anti-SARS-CoV S protein (Similar to 240C) SARS coronavirus].

Antiviral Drug Study

Vero E6 cells, HEK293-ACE2 and hiPSC-CM cells were seeded on 96-well plates and were pretreated with drugs for one to twenty four hours, then SARS-CoV-2 inoculum (MOI 0.1) was added. For ALI cultures, the drug was added in the basal chamber. For DMSO vehicle treated cells, with or without viral infections, were included as controls. 48 hpi, the cells were fixed and immunostained with anti-dsRNA antibody (J2 clone; Absolute Antibody Inc, USA) or anti-spike antibody (NR-616 Monoclonal Antibody) to assess virus replication.

Viral Titer by TCID50 (Median Tissue Culture Infectious Dose) assay

Viral production by infected cells was measured by quantifying TCID50 as previously described (Gauger and Vincent, 2014). Briefly, Vero E6 cells were plated in 96-well plates at a density of 5 x10^3cells/well. The next day, culture media samples collected from ALI at various time points were subjected to 10-fold serial dilutions ($10^1$to 10^6) and inoculated onto Vero E6 cells. The cells were incubated at 37°C with 5% CO2. After 3 to 4 days, each inoculated well was evaluated for presence or absence of viral CPE and percent infected dilutions immediately above and immediately below 50% were determined. TCID50 was calculated based on the method of Reed and Muench.

Cytotoxicity Assay

We performed MTT ((3-(4, 5-dimethylthiazolyl-2)-2,5-diphenyltetrazolium bromide) Cell Proliferation assay (ATCC) as indicated by manufacture. Vero E6 cells were seeded on 96-well plates. After 48 hours of drug treatment, MTT reagent (10 ul) was added to the cells and incubate for 4 hours at 37°C. The detergent reagent (100 ul) was then added for 2 hours to the cells and incubated at room temperature for 2 hours. Absorbance of each well was recorded and triplicate values of each condition was measured. Percent cytotoxicity for each compound was calculated based on vehicle (DMSO) treated cells.

Viral replication kinetics

All Calu-3 and A549-ACE2 experiments with live CoVs were performed under BSL-3 conditions at the Heinrich Pette Institute (Hamburg, Germany) following standard operating procedures. Calu-3 cells were seeded in 24-well plates with 3.5 × 105 cells/ ml, 1 mL per well, for 24 h. The compounds to be tested were diluted in CoV infection medium to reach the final concentrations. The growth medium was removed from the cells, cells were washed once with 1x PBS (phosphate buffered saline), and subsequently inoculated with either SARS-CoV-2 (SARS-CoV-2/Germany/HPI06-n/2020), SARSCoV or MERS-COV at a MOI (multiplicity of infection) of 0.01. After attachment of viral particles to the cells for 45 min, the inoculum was removed, cells were washed twice with 1x PBS, and infection medium containing compounds was added (1 ml/well). As CoV replication peaks at approximately 48 h post infection (p.i.; data not shown), this time point was chosen for all subsequent analyses. At 48 h p.i., supernatants were collected from infected cells and stored at −80°C. Then, viral titers were determined by plaque test on Vero E6 cells as described below.

A549 cells stably expressing ACE2 were seeded into 96-well plates. On the next day, 3-fold serial dilutions of given drugs were added to the cells covering a drug concentration range of 2.5 nM - 50 μM for Berzosertib, and 0.5 nM - 10 μM for remdesivir. Thereafter, cells were inoculated with SARS-CoV-2 (MOI = 1) and 24 h later, cells were fixed and viral nucleocapsid was detected by immunostaining using a secondary antibody that was coupled to horseradish peroxidase. Bound secondary antibody was detected by colorimetric assay and signal was quantified by measuring absorbance at 405 nm. Values were normalized using solvent control (0.5% DMSO) with or without virus infection. Synergistic effect of berzosertib and remdesivir in infected A549-ACE2 cells are investigated as follows: A549-ACE2 cells were treated with a mixture of berzosertib and remdesivir (concentrations specified in the top and bottom, respectively) prepared in a 7-by-7 concentration matrix, which generated 49 combinations ranging from 0.014 μM - 10 μM for berzosertib, and 6.8 nM - 5 μM for remdesivir. Cells were inoculated with SARS-CoV-2 and antiviral activity was measured by in-well immunostaining as described above.

Plaque test

Viral titers in supernatants collected from CoV infected cells were determined by plaque test on Vero E6 cells. Briefly, Vero E6 cells were seeded in 12-well plates (1:6 dilution of a confluentflask), 1.5 ml/well, for 24 h. Cell culture supernatants were 10-fold serially diluted in 1x PBS. The growth medium was removed from the cells, cells were washed once with 1x PBS, anddiluted supernatants were added (150 μl/well). After 30 min inoculation, an overlay medium (double-concentrated minimal essential medium (MEM; supplemented with 2 % L-glu, 2 % P/S, 0.4 % bovine serum albumin (BSA)), mixed 1:1 with 2.5 % avicel solution (prepared in ddH2O)) was added to the cells (1.5 ml/well). Then, cells were incubated for 72 h at 37°C. After 72 h, the overlay medium was removed from the cells, and following a washing step with 1x PBS the cells were fixed with 4 % paraformaldehyde (PFA) for at least 30 min at 4°C. Subsequently, the 4 % PFA solution was removed, and the cells were counterstained with crystal violet solution to visualize the virus-induced plaques in the cell layer. The number of plaques at a given dilution was used to calculate the viral titers as plaque-forming units (PFU/ml).

Cell viability assay

Calu-3 and A549-ACE2 were seeded in 96-well plates with 3.5 × 10^5 cells/ml, 100 μL per well, for 24 h. The compounds to be tested or pure DMSO as positive control were serially diluted in CoV infection medium (DMEM, supplemented with 1 % L-glu, 1 % P/S and 2 % FBS) to obtain 5-fold of the desired final concentrations. The growth medium was removed from the cells and

replaced with 80 μl/well of fresh infection medium. Subsequently, 20 μL of the diluted compounds were added in quadruplicates for each concentration (i.e., 5-fold dilution to reach the final concentrations). Cells were incubated for 48 h at 37°C (5% CO_2, 96 % rH). At 48 h post treatment, cell viability was measured on a Tecan Safire 2 plate reader using the CellTiter 96® Non-Radioactive Cell Proliferation Assay (MTT) (Promega) according to manufacturer's instructions.

High-content screening assay

Compounds were acoustically transferred into 384-well μclear-bottom plates (Greiner, Part. No. 781090-2B). HeLa-ACE2 cells were seeded in 13 μL DMEM with 2% FBS at a density of 1.0×10^3 cells per well. Plated cells were transported to the BSL3 facility where 13 μL of SARS-CoV-2 diluted in assay media was added per well at a concentration of 2.0×10^6 PFU/mL (assay multiplicity of infection (MOI) = 0.65). Plates were incubated for 24 hours at 34°C 5% CO_2, and then fixed with 25 μL of 8% formaldehyde for 1 hour at 34°C 5% CO_2. Plates were washed with 1xPBS 0.05% Tween 20 in between fixation and subsequent primary and secondary antibody staining. Human polyclonal sera diluted 1:500 in Perm/Wash buffer (BD Biosciences 554723) was added to the plate and incubated at room temperature for 2 hours. 6 μg/mL of goat anti-human H^+L conjugated Alexa 488 (Thermo Fisher Scientific A11013) together with 8 μM of antifade-46-diamidino-2-phenylindole (DAPI; Thermo Fisher Scientific D1306) in SuperBlock T20 (PBS) buffer (Thermo Fisher Scientific 37515) was added to the plate and incubated at room temperature for 1 hour in the dark. Plates were imaged using the ImageXpress Micro Confocal High-Content Imaging System (Molecular Devices) with a 10 × objective, with 4 fields imaged per well. Images were analyzed using the Multi-Wavelength Cell Scoring Application Module (MetaXpress), with DAPI staining identifying the host-cell nuclei (the total number of cells in the images) and the SARS-CoV-2 immunofluorescence signal leading to identification of infected cells.

HeLa-ACE2 Uninfected host cell cytotoxicity counter screen

Compounds were acoustically transferred into 1,536-well μclear plates (Greiner Part. No. 789091). HeLa-ACE2 cells were maintained as described for the infection assay and seeded in the assay-ready plates at 400 cells/well in DMEM with 2% FBS and plates were incubated for 24 hours at 37°C 5% CO_2. To assess cell viability, the Image-iT DEAD green reagent (Thermo Fisher) was used according to manufacturer instructions. Cells were fixed with 4% paraformaldehyde, and counterstained with DAPI. Fixed cells were imaged using the ImageXpress Micro Confocal High-Content Imaging System (Molecular Devices) with a 10 × objective, and total live cells per well quantified in the acquired images using the Live Dead Application Module (MetaXpress).

Data analysis of SARS-CoV-2/HeLa-ACE2 Experiments

Image analysis was carried out with MetaXpress (version 6.5.4.532). Primary *in vitro* screen and the host cell cytotoxicity counter screen data were uploaded to Genedata Screener, Version 16.0.3-Standard. Data were normalized to neutral (DMSO) minus inhibitor controls (2.5 μM remdesivir for antiviral effect and 10 μM puromycin for infected host cell toxicity). For the uninfected host cell cytotoxicity counter screen 40 μM puromycin (Sigma) was used as the positive control. For dose response experiments compounds were tested in technical triplicates on different assay plates and dose curves were fitted with the four parameter Hill Equation. Technical replicate data were analyzed using median condensing.

Image Analysis/Quantification

Microscope images were obtained using the Leica DM IRB and Zeiss Software Program. Three to five images per well were quantified for each condition using ImageJ's plugin Cell Counter feature was used to count the positively stained cells by a double blinded approach.

Immunohistochemistry

Cells were fixed with methanol (incubated in −20°C freezer until washed with PBS) or 4% Paraformaldehyde for 30-60 minutes. Cells were washed 3 times with 1x PBS and permeabilized using blocking buffer (0.3% Triton X-100, 2% BSA, 5% Goat Serum, 5% Donkey Serum in 1 X PBS) for 1 hour at room temperature. For immunostaining, cells were incubated overnight at 4°C with each primary antibody. The cells were then washed with 1X PBS three times and incubated with respective secondary antibody for 1 hour at room temperature. Nuclei were stained with DAPI (4',6-Diamidino-2-Phenylindole, Dihydrochloride) (Life Technologies) at a dilution of 1:5000 in 1 X PBS. Image acquisition was done using Leica DM IRB fluorescent microscopes.

RNA sample preparation and RT-qPCR

To determine levels of SARS-CoV-2 virus in cells, total RNA was extracted using RNeasy Mini Kit (QIAGEN), as per the manufacturer's instructions. RNA was quantified using a NanoDrop 1,000 Spectrophotometer (Thermo Fisher Scientific). cDNA was prepared from 1 μg of RNA using random hexamer primers and the SuperScript III Reverse Transcriptase Kit (Thermo Fischer Scientific). QPCR was performed using SSOAdvanced Universal SYBR Green Supermix (Bio-Rad) using a CFX384 Touch Real-Time PCR Detection System (Bio-Rad). Briefly, amplification was performed using 10 μL volume reactions in a 384-well plate format with the following conditions: 95°C for 30 s for polymerase activation and cDNA denaturation, then 40 cycles at 95°C for 15 s and 60°C for 1 minute, with a melt-curve analysis at 65-95°C and 0.5°C increments at 2-5 s/step. The relative concentration of each transcript was calculated using the $2^{-\Delta CT}$ method and Glyceraldehyde 3-phosphate dehydrogenase (GAPDH) threshold cycle (C_T) values were used for normalization.

The qPCR primer pairs for mRNA transcript targets are provided in the Key resources table. . SARS-CoV-2 RNA transcript levels were quantified by comparing them to a standard curve generated using serial ten-fold dilutions (10^1-10^9 copies) of a SARS-CoV-2 N gene containing plasmid. SARS-CoV-2 RNA levels were expressed as SARS-CoV-2 genome copies per 1 μg of RNA using the standard curve.

Western Blot analysis

Cells were lysed in 50 mM Tris pH 7.4, 1% NP-40, 0.25% sodium deoxycholate, 1 mM EDTA, 150 mM NaCl, 1 mM Na3VO4, 20 Mm or NaF, 1mM PMSF, 2 mg ml^{-1} aprotinin, 2 mg ml^{-1} leupeptin and 0.7 mg ml^{-1} pepstatin or Laemmli Sample Buffer (Bio Rad, Hercules, CA). Cell lysates were resolved by SDS-PAGE using 10% gradient gels and transferred to a 0.2 μm PVDF membrane. Subsequently, the membranes were blocked with 5% skim milk and 0.1% Tween-20 in 1x TBST (0.1% Tween-20) at room temperature for 1 hour. The membranes were then probed with respective monoclonal antibodies and detected by Thermo Scientific SuperSignal West Femto Maximum Sensitivity Substrate.

QUANTIFICATION AND STATISTICAL ANALYSIS

IC_{50} values were obtained by fitting a sigmoidal curve onto the data of an eight point dose response curve experiment. Isobologram combinatorial data were analyzed for inhibitory, additive or synergistic effects by using the Compusyn software package (Combo-Syn. Inc.) (Chou, 2006). All testing was done at the two-sided alpha level of 0.05. Data were analyzed for statistical significance using unpaired Student's t test to compare two groups (uninfected versus infected) or a non-parametric t test (Mann-Whitney Test) with Graph Pad Prism software, version 8.1.2 (GraphPad Software, US).

Cell Reports, Volume *35*

Supplemental information

Antiviral drug screen identifies

DNA-damage response inhibitor

as potent blocker of SARS-CoV-2 replication

Gustavo Garcia Jr., Arun Sharma, Arunachalam Ramaiah, Chandani Sen, Arunima Purkayastha, Donald B. Kohn, Mark S. Parcells, Sebastian Beck, Heeyoung Kim, Malina A. Bakowski, Melanie G. Kirkpatrick, Laura Riva, Karen C. Wolff, Brandon Han, Constance Yuen, David Ulmert, Prabhat K. Purbey, Phillip Scumpia, Nathan Beutler, Thomas F. Rogers, Arnab K. Chatterjee, Gülsah Gabriel, Ralf Bartenschlager, Brigitte Gomperts, Clive N. Svendsen, Ulrich A.K. Betz, Robert D. Damoiseaux, and Vaithilingaraja Arumugaswami

SUPPLEMENTARY FIGURES

Figure S1

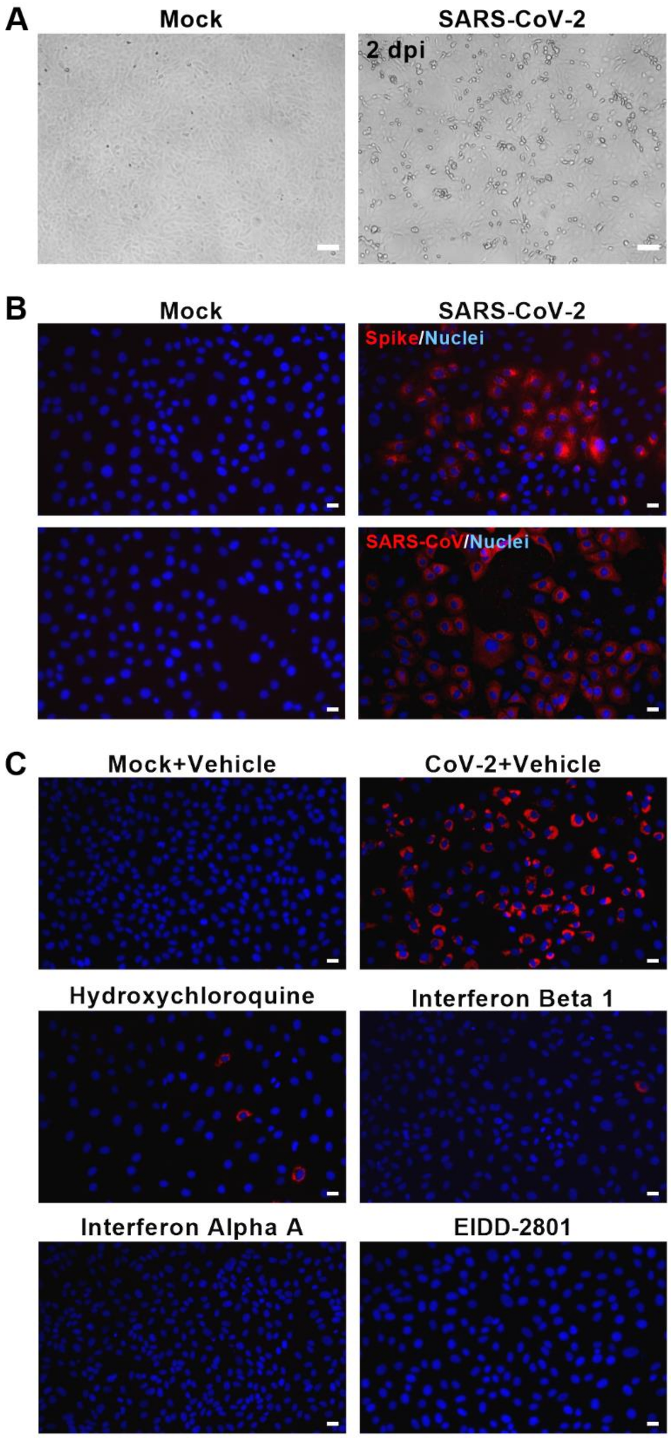

Figure S1. Infectious SARS-CoV-2 cell culture system. (A) Bright field images of Vero E6 cells infected with SARS-CoV-2 virus. Viral cytopathic effects (CPE) are noted in the infected culture. Scale Bar: 50 µm. (B) IFA images of infected cells (48 hpi) stained for SARS-CoV-2. Mouse monoclonal antibody (MS Ab) targeting Spike and a Guinea pig polyclonal SARS-CoV antibody were used. Mock infected cells were included as negative control. Scale Bar: 25 µm. (C) IFA images show compounds with antiviral activity. Images depict that hydroxychloroquine, interferons, and EIDD-2801 (Molnupiravir) (10µM) are effective in blocking SARS-CoV-2 infection. The mock and infected cells were immunostained with dsRNA antibody, which recognizes double stranded genomic RNA generated during viral replication. 20X magnification. Representative data from three or more independent experiments are presented. Related to Figure 1.

Figure S2

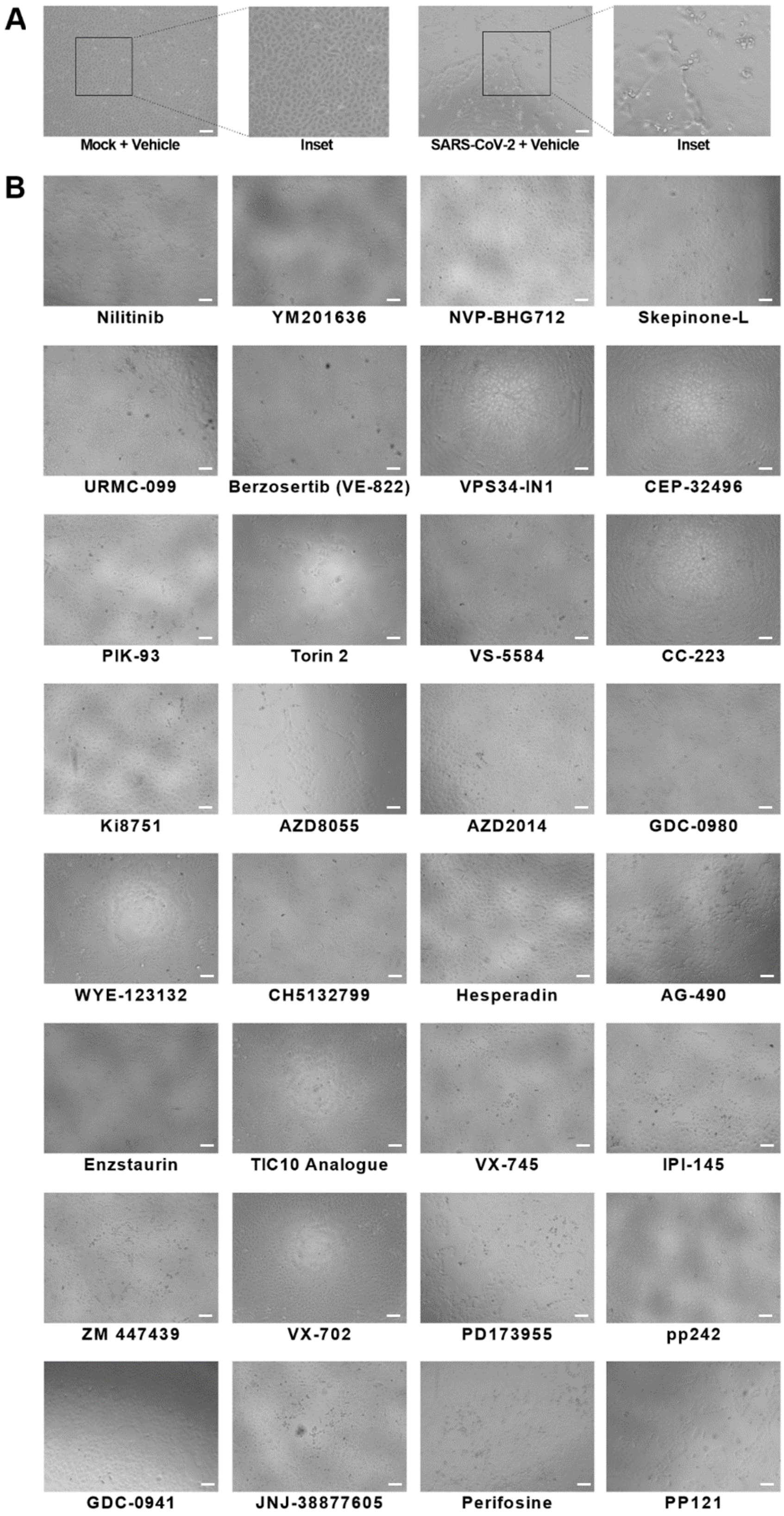
A
Mock + Vehicle
Inset
SARS-CoV-2 + Vehicle
Inset
B
Nilitinib
YM201636
NVP-BHG712
Skepinone-L
URMC-099
Berzosertib (VE-822)
VPS34-IN1
CEP-32496
PIK-93
Torin 2
VS-5584
CC-223
Ki8751
AZD8055
AZD2014
GDC-0980
WYE-123132
CH5132799
Hesperadin
AG-490
Enzstaurin
TIC10 Analogue
VX-745
IPI-145
ZM 447439
VX-702
PD173955
pp242
GDC-0941
JNJ-38877605
Perifosine
PP121

Figure S2. Primary screen of compounds inhibiting SARS-CoV-2 viral cytopathic effect. (A) DMSO Vehicle treated Vero E6 cells with SARS-CoV-2 infection had pronounced viral CPE at 48 hpi. Uninfected cells (Mock+vehicle) are included as negative control. Scale Bar: 50 µm. (B) Bright field microscopic images of drug compounds treated SARS-CoV-2 infected cells showing no or reduced level of viral CPE. Scale Bar: 50 µm. Related to Figure 1.

Figure S3

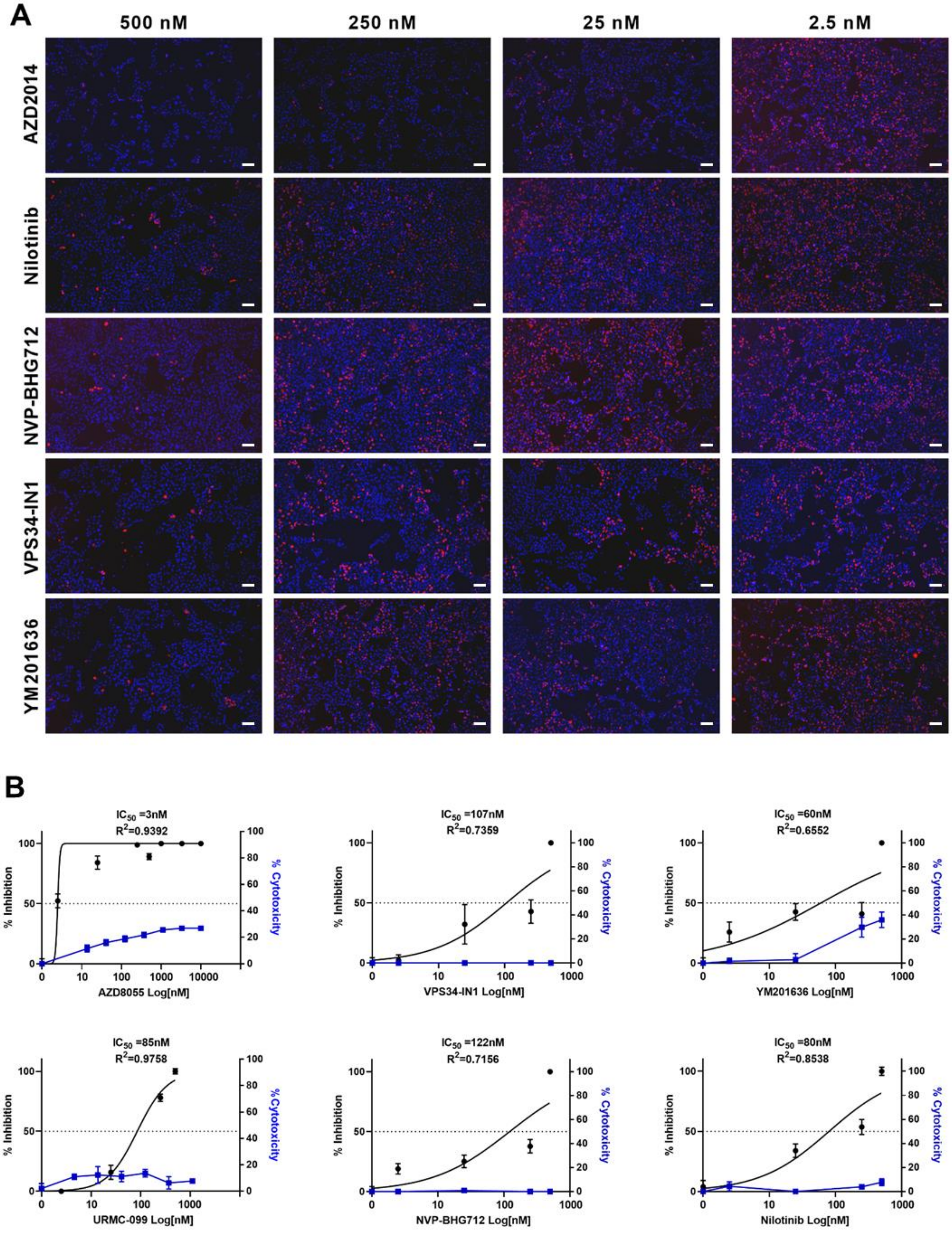
A
500 nM
250 nM
25 nM
2.5 nM
AZD2014
Nilotinib
NVP-BHG712
VPS34-IN1
YM201636
B
IC50 =3nM
R2=0.9392
AZD8055 Log[nM]
IC50 =107nM
R2=0.7359
VPS34-IN1 Log[nM]
IC50 =60nM
R2=0.6552
YM201636 Log[nM]
IC50 =85nM
R2=0.9758
URMC-099 Log[nM]
IC50 =122nM
R2=0.7156
NVP-BHG712 Log[nM]
IC50 =80nM
R2=0.8538
Nilotinib Log[nM]
% Inhibition
% Cytotoxicity

Figure S3. Secondary Screen in Vero E6 cells. (A) Immunofluorescent images of SARS-CoV-2 (red) infected Vero E6 cells treated with indicated drug compounds at various concentrations. (B) Graphs show percent inhibition of SARS-CoV-2 infectivity by indicated compounds. Note: IC50 of each compound is shown in the graph. Related to Figure 1.

Figure S4

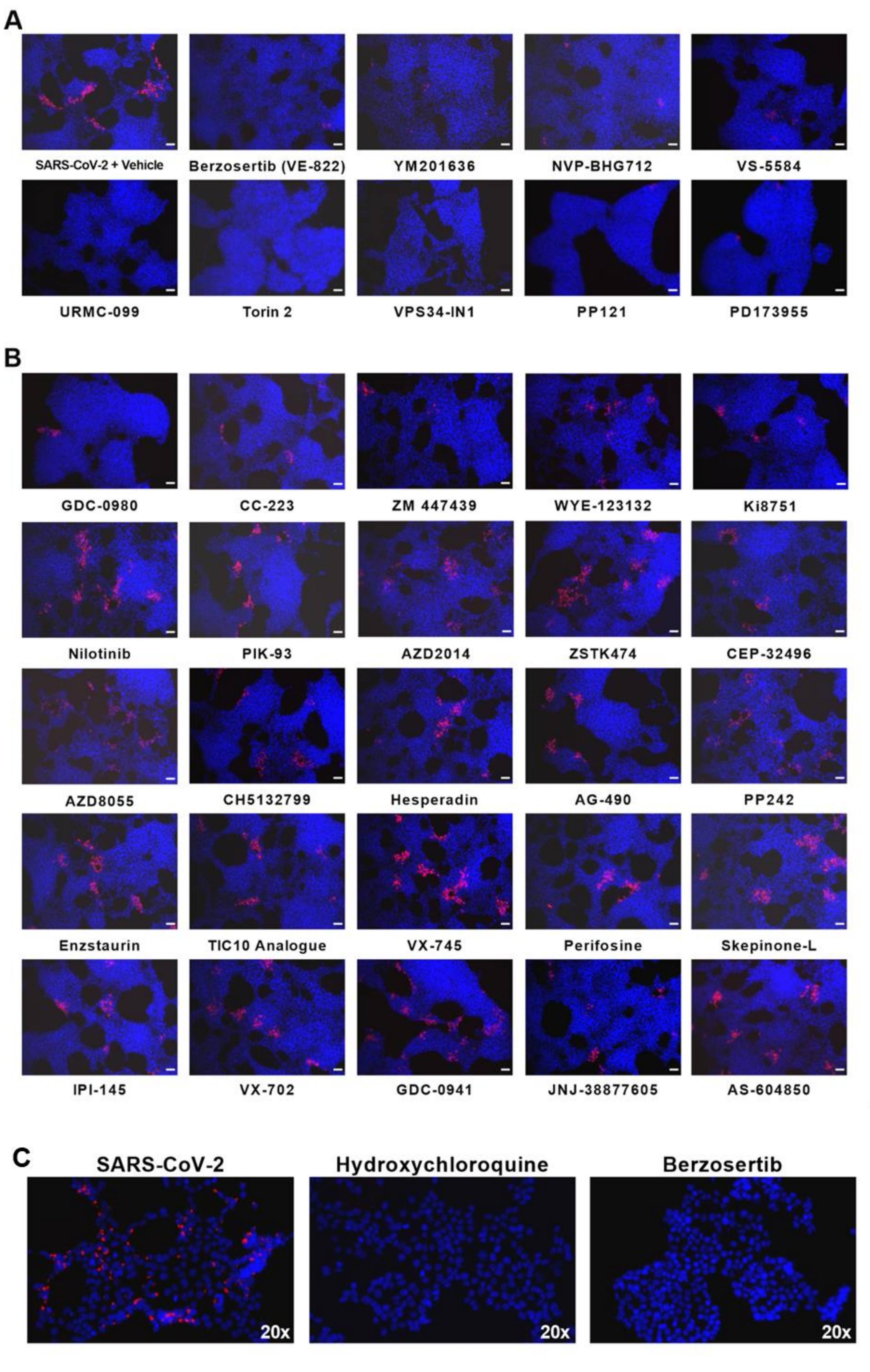
A
SARS-CoV-2 + Vehicle
Berzosertib (VE-822)
YM201636
NVP-BHG712
VS-5584
URMC-099
Torin 2
VPS34-IN1
PP121
PD173955
B
GDC-0980
CC-223
ZM 447439
WYE-123132
Ki8751
Nilotinib
PIK-93
AZD2014
ZSTK474
CEP-32496
AZD8055
CH5132799
Hesperadin
AG-490
PP242
Enzstaurin
TIC10 Analogue
VX-745
Perifosine
Skepinone-L
IPI-145
VX-702
GDC-0941
JNJ-38877605
AS-604850
C
SARS-CoV-2
Hydroxychloroquine
Berzosertib
20x
20x
20x

Figure S4. Secondary Screen in HEK293-ACE2 cells. The cells were pre-treated with each of the 34 compounds (250 nM) from the primary screen for 24 hours. The following day the cells were infected with SARS-CoV-2 with an MOI of 0.1. At one day post-infection the cells were fixed in methanol and immunostained to detect SARS-CoV-2 Spike antigen (red). (A) Immunofluorescent images of compounds having potent anti- SARS-CoV-2 activity are shown. Vehicle control with infection was included as reference. (B) Drugs with low or no anti-viral activity at tested dose of 250 nM are presented. Note: Compounds including nilotinib and AZD2014 had not exhibited potent anti-viral activity at tested 250 nM dose level. Scale Bar=25um. (C) IFA images show SARS-CoV-2 (red) infection in untreated cells and hydroxychloroquine (10 µM) treated cells. Complete inhibition of SARS-CoV-2 infection in berzosertib (100nM) treated cells is noted. 20x magnification. Related to Figure 1.

Figure S5

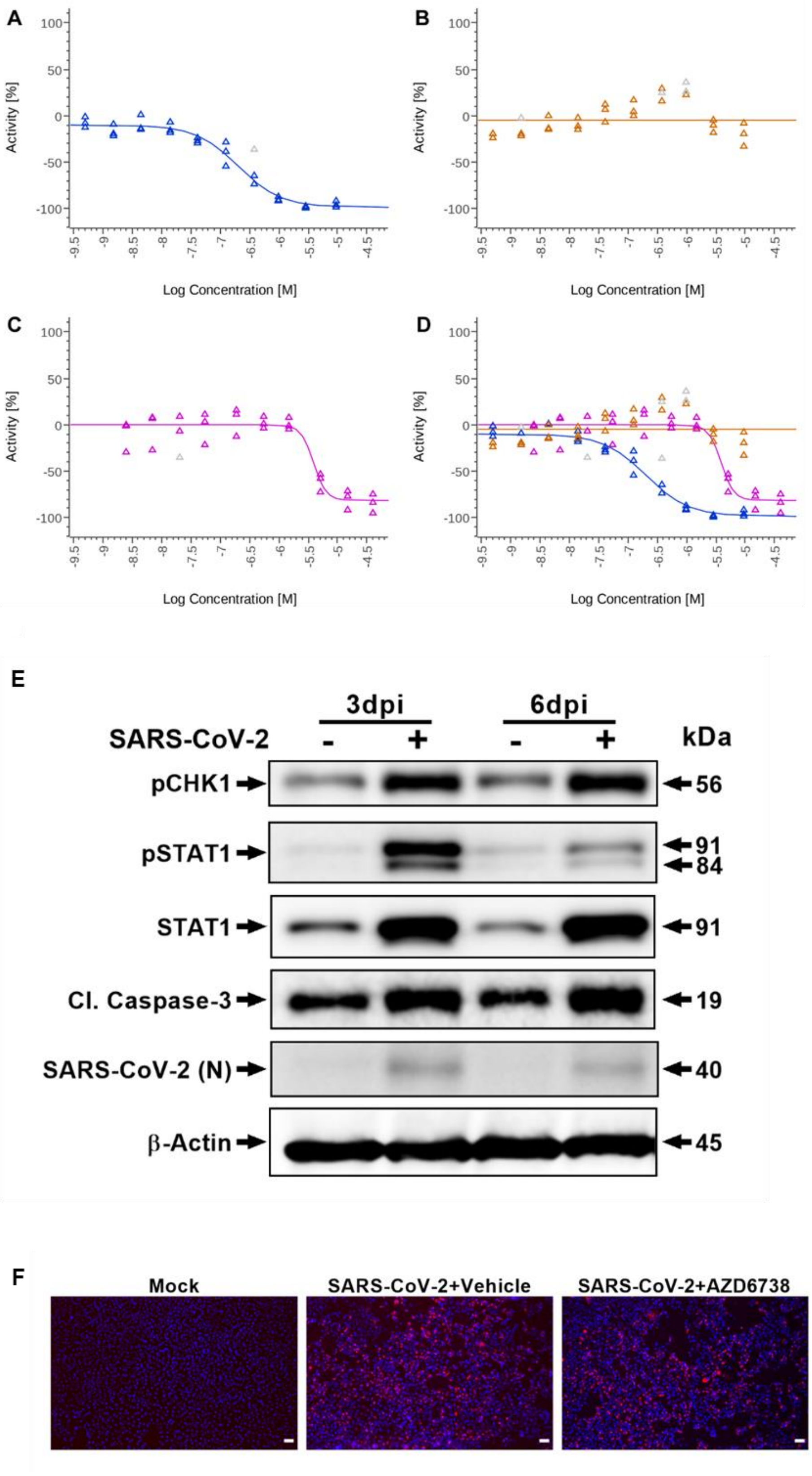
A
B
C
D
Activity [%]
Log Concentration [M]
100
50
0
-50
-100
-9.5
-9
-8.5
-8
-7.5
-7
-6.5
-6
-5.5
-5
-4.5
E
3dpi
6dpi
SARS-CoV-2
-
+
-
+
kDa
pCHK1
56
pSTAT1
91
84
STAT1
91
Cl. Caspase-3
19
SARS-CoV-2 (N)
40
β-Actin
45
F
Mock
SARS-CoV-2+Vehicle
SARS-CoV-2+AZD6738

Figure S5. Berzosertib inhibits SARS-CoV-2 replication in human HeLa-ACE2 cells and affects DDR pathway. (A) Graph shows antiviral activity measured with a SARS-CoV-2 immunofluorescence signal leading to identification of infected cells with 0% activity equals 100% infected cells. (B) total cells per well in SARS-CoV2 infected cell test with 0% activity equaling no change vs. control (C) total cells per well in HeLa-ACE2 uninfected cell control. (D) Overlay of curves in A, B and C. (E) SARS-CoV-2 activates DDR pathway by CHK1 phosphorylation. Western blot analysis shows phosphorylation of key ATR kinase downstream target protein CHK1 in SARS-CoV-2 infected lung proximal airway epithelial cells grown in air-liquid interface (ALI) culture (48hpi). (F) Evaluating additional DNA-Damage Pathway ATR Kinase Inhibitor. Vero E6 cells pre-treated (24 hours) with AZD6738 (10µM) were infected with SARS-CoV-2 48 hours post-infection virus replication was visualized with immunostaining. Scale Bar=100 µm. IFA images show SARS-CoV-2 (red) infection in vehicle or AZD6738 treated cells. No inhibition of SARS-CoV-2 infection in AZD6738 (10µM) treated cells is noted. Related to Figure 3 and 4.

SUPPLEMENTARY TABLE

Table S2. Drugs compounds selected from primary screen having antiviral activity at 250 nM concentration (Related to Figure 1).

	**Compound Name**	**Activity**
1	Berzosertib (M6620)	ATR kinase inhibitor
2	Nilotinib (AMN-107)	Bcr-Abl inhibitor
3	NVP-BHG712	EphB4 inhibitor
4	VPS34-IN1	Vps34 inhibitor
5	YM201636	PIKfyve inhibitor
6	AZD-2014 (Vistusertib)	Inhibitor mTOR and multiple PI3K isoforms (α/β/γ/δ)
7	AZD8055	ATP-competitive mTOR inhibitor
8	VS-5584 (SB2343)	Dual PI3K/mTOR inhibitor
9	Torin 2	Selective mTOR inhibitor
10	CC-223 (Onatasertib)	mTOR inhibitor
11	WYE-125132 (WYE-132)	ATP-competitive mTOR inhibitor
12	PP242 (Torkinib)	mTOR inhibitor
13	ZSTK474	Inhibitor of class I PI3K isoforms
14	GDC-0941 (Pictilisib)	PI3Kα/δ inhibitor
15	GDC-0980 (RG7422)	Class I PI3K inhibitor for PI3Kα/β/δ/γ
16	AS-604850	ATP-competitive PI3Kγ inhibitor
17	CH5132799	Inhibitor of class I PI3Ks
18	IPI-145 (INK1197)	selective PI3K δ/γ inhibitor
19	PIK-93	PI3Kγ and PI4KIIIβ inhibitor
20	Enzastaurin (LY317615)	PKCβ inhibitor
21	TIC10 Analogue	Inactivates Akt and ERK
22	Perifosine (KRX-0401)	Akt inhibitor
23	AG-490 (Tyrphostin B42)	EGFR and JAK2 inhibitor
24	VX-745	p38α MAPK inhibitor
25	Skepinone-L	p38α-MAPK inhibitor
26	VX-702	p38α MAPK inhibitor
27	CEP-32496	Inhibitor of BRAF(V600E/WT) and c-Raf
28	ZM 447439	ATP-competitive inhibitor for Aurora kinases A and B
29	Hesperadin	Aurora kinase B inhibitor
30	JNJ-38877605	ATP-competitive inhibitor of c-Met
31	Ki8751	VEGFR2 inhibitor
32	URMC-099	Mixed lineage kinase (MLK) inhibitor
33	PD173955	Bcr-Abl inhibitor
34	PP121	Inhibitor of PDGFR, Hck, mTOR, VEGFR2, Src and Abl
